# Investigation of N-(2-oxo-2H-chromen-3-carbonyl)cytisine’s Molecular Structure in Solution

**DOI:** 10.3390/molecules30204139

**Published:** 2025-10-20

**Authors:** Kymbat Kopbalina, Aigerim Adekenova, Zhanar Shaimerdenova, Zhanargul Kairatova, Kuanysh Shakarimova, Dmitrii Pankin, Mikhail Smirnov, Anarkul Kishkentayeva, Makpal Artykbayeva, Roza Jalmakhanbetova

**Affiliations:** 1 Department of Physics and Nanotechnology, Buketov Karaganda University, Universitetskaya 28, Karaganda 100024, Kazakhstan; kymbatkargtu@gmail.com; 2 School of Pharmacy, Karaganda Medical University, Karaganda 100012, Kazakhstan; aika_as.87@mail.ru (A.A.); arsenzhan@bk.ru (Z.S.); zhanargul.kayratova01@gmail.com (Z.K.); ladyjoy82@mail.ru (K.S.); 3 Center for Optical and Laser Materials Research, St. Petersburg State University, Ulianovskaya 5, 198504 St. Petersburg, Russia; dmitrii.pankin@spbu.ru; 4 Faculty of Physics, St. Petersburg State University, Universitetskaya Nab. 7/9, 199034 St. Petersburg, Russia; m.smirnov@spbu.ru; 5 Department of Chemistry, Faculty of Natural Sciences, L.N. Gumilyov Eurasian National University, Astana 010000, Kazakhstan; artykbayevamakpal@gmail.com

**Keywords:** cytisine, coumarine, NMR spectroscopy, UV-Vis absorbance, density functional theory, conformer

## Abstract

Cytisine and coumarin derivatives are promising for the creation of new drugs with antiarrhythmic, antiepileptic, antidiabetic, anti-inflammatory, and antimicrobial effects. In this study, the molecular structure of the cytisine and coumarin derivative in solution, a recently synthesized substance N-(2-oxo-2H-chromen-3-carbonyl)cytisine, was studied by NMR and UV-Vis absorption spectroscopies accompanied by a theoretical study based on density functional theory. The existence of four stable conformers associated with the rotation of the cytisine part relative to the coumarin part due to a sufficiently flexible intermediate part has been demonstrated. Their energy and concentrations were estimated. In the 1H and 13C NMR spectra, peaks were found that correspond to individual conformers and groups of conformers. The UV-visible absorption spectrum also revealed spectral features associated with different conformers. It was shown that the obtained results are consistent with earlier studies about conformational state identification in cytisine derivatives functionalized with flexible parts. The obtained theoretical and experimental results provide useful spectroscopic information for such conformer identification in this and structurally similar substances.

## 1. Introduction

The molecular hybridization approach [1,2] allows combining the advantages of several compounds at once. Therefore, it is one of the useful approaches for the synthesis of new substances promising for pharmacological needs. The object of this study is the complex molecule (hereinafter simply ‘complex’) N-(2-oxo-2H-chromen-3-carbonyl)cytisine synthesized earlier in [3], which consists of a coumarin moiety, an intermediate (linking) part, and a cytisine moiety. The potential prospects of this complex are associated with the biological activity of its individual moieties.

The coumarin itself and its derivatives have pronounced luminescent properties. Combined with their diverse biological activities, including, among others, anticancer, anticoagulant, and antidiabetic properties [4,5,6,7], this makes them outstanding candidates for the synthesis of drugs whose biological action can be observed by luminescence imaging while monitoring the spectral response (wavelength, band intensity, and bandwidth), which is sensitive to the chemical structure.

The natural alkaloid cytisine plays an important role due to its binding to neuronal nicotinic acetylcholine receptors (nAChRs) [8]. This influences fundamental processes in the central nervous system [8] and creates the potential to reduce nicotine and alcohol addiction [9,10]. Understanding the possible structural states of a molecule that contains cytisine as a moiety is important because the structure and stereochemistry of a molecule are closely related to the biological activity it exhibits [11,12]. As noted in previous studies, conformational diversity is possible in cytisine derivatives, associated with rotation of the cytisine part as a whole relative to the rest of the molecule and formation of intermolecular hydrogen bonds and contacts [11,13,14,15]. At the same time, the structure of cytisine and its derivative will be significantly affected by both intramolecular [11] and intermolecular [16] hydrogen bonds. The latter play a significant role in the structure of the molecule in the crystalline state, which structure may differ from the structure of the molecule in solution [15]. To study the structure of the cytisine molecule and its derivatives in solution, such experimental approaches as NMR spectroscopy [13,14,15], mass spectrometry [13,17], and electron absorption spectroscopy [13,18] in the UV-visible (UV-Vis) range and luminescence [18] are often used. In this case, both non-polar (e.g., n-hexane) [19] and polar solvents (e.g., dimethyl sulfoxide (DMSO), chloroform, acetonitrile) [11,14,18] were used in various studies. To interpret the obtained results, theoretical studies of varying levels of rigor were conducted; see, for example, Refs [11,14,18]. DMSO and ethanol were chosen as solvents in this work. The choice of the DMSO solvent is related to the practice of using this solvent in studying the molecular structure by NMR spectroscopy; the ethanol solvent is closer to a solvent that can be used for pharmacological purposes.

In light of the above, it seems appropriate to study the conformational states of the coumarin–cytisine complex by using 13C NMR and 1H NMR spectroscopy and UV-Vis absorption spectroscopy. The NMR spectroscopy method was chosen due to its sensitivity to the structure of the substance, consisting of the sensitivity of NMR signals from carbon and hydrogen atoms to their immediate environment. Moreover, unlike vibrational spectroscopy methods, the contribution of the solvent to NMR spectra is relatively small. Another advantage of the NMR spectroscopy method is the possibility of spatial localization of the source of the recorded signal. The NMR signals can be associated with specific atoms, while in vibrational spectra, vibrational modes can be delocalized within a group of atoms. In addition to the technically complex method of NMR spectroscopy, UV-visible electron absorption spectroscopy was used to study the substance in solution. This method is much simpler to implement and can therefore be used as a basis for fast, in-line measurements carried out in industrial production. To interpret the spectral data and establish the structure–spectrum correlation, the corresponding quantum chemical calculations were also performed within the framework of this work.

Thus, this article presents the results of theoretical and experimental studies of the conformational states of the N-(2-oxo-2H-chromene-3-carbonyl)cytisine complex with a flexible intermediate part between the cytisine and coumarin fragments in order to establish a structure–spectrum correlation. The theoretically predicted structures and experimentally observed manifestation of conformational states are discussed.

## 2. Results and Discussions

As in the Ref. [3], we consider the molecular complex consisting of three parts: coumarin, cytisine, and intermediate moieties. The flexibility of the intermediate part can lead to different orientations of the coumarin and cytisine parts relative to each other. As a result of such adjustment, in the initial approximation it can be considered that the geometry of the rings in the cytisine and coumarin parts remains virtually unchanged. Based on this, it is proposed to classify the resulting conformers based on the mutual arrangement of the polar C=O bonds in different parts, more precisely based on the dihedral angle of the type O_m_=C_m_C_n_=O_n_, where m and n subindexes refer to coumarin, cytisine, and intermediate moieties.

**Figure 1:**
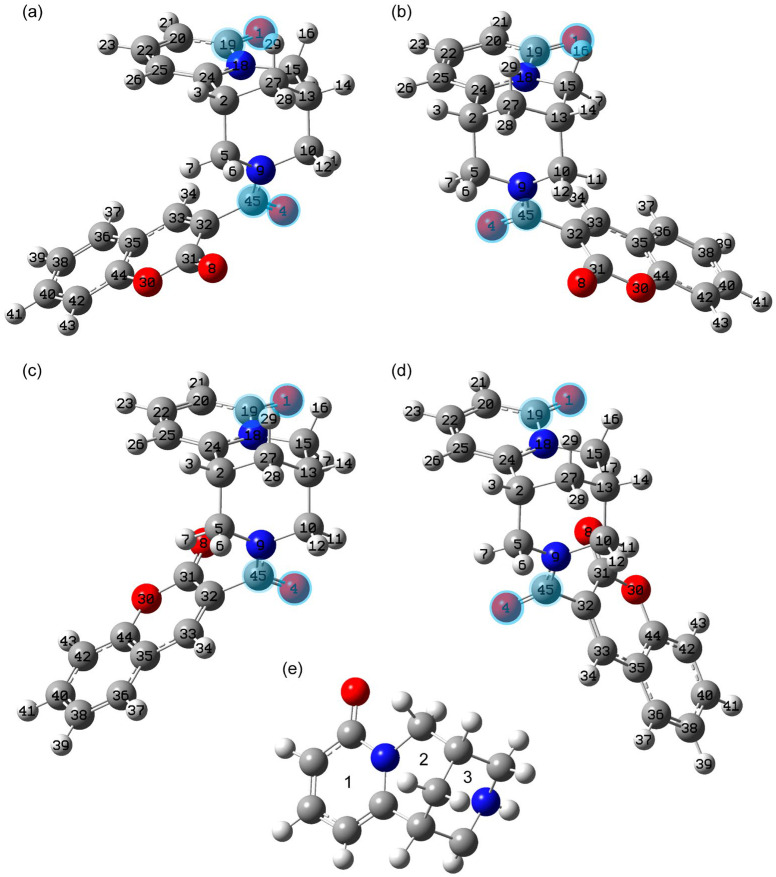
Structures of conformers 1 (**a**), 2 (**b**), 3 (**c**), and 4 (**d**) optimized at the B3LYP/6-311G(2d,p) level with the DMSO solvent influence taken implicitly via the polarizable continuum model (see the details in Section 3.4) and rings numbered in cytisine moiety (**e**). The carbon, nitrogen, oxygen, and hydrogen atoms are dark gray, blue, red, and light gray, respectively. The demonstrated atom labels are used further in the text and tables. The C=O bonds forming dihedral angles discussed in the text are highlighted.

In the first stage, a search for conformers was conducted using molecular dynamics simulations with varying dihedral angles. Details are provided in Section 3.4. The non-optimized and total energies for the 6000 lowest states are demonstrated in Figure S1a. The geometries were subjected to the optimization. It was found that various geometries with various energies become degenerated to the six main low energy levels (energy states during force field optimizations) demonstrated in Figure 1b. The example of six lowest energy optimized geometries with various energy levels is demonstrated in Figure S2.

In the second stage, the DFT optimization was performed, establishing how conformers interconvert into each other. One of the types of conformers suggested by molecular dynamics demonstrated imaginary frequency on the basis of DFT calculations (see Figure S3). It corresponds to the 1st-order saddle point at the potential energy surface. It was excluded. In this article, the discussion is devoted to the rotational conformers. They were obtained as a result of relaxed potential energy scans performed while rotating around single bonds in the intermediate part. We began by changing the C5N9C45C32 dihedral angle (see Figure S4a), which led to conformer 2, shown in Figure 1b. Two potential energy scans were then performed (see Figure 1b,c) with changes in the C31C32C45N9 dihedral angle. These resulted in conformers 3 and 4. The optimized geometries of the found stable conformers are demonstrated in Figure 1 and given in Table S1.

For ease of presentation, we have introduced the numbering of the rings in the cytisine parts (see Figure 1e). For brevity, the number of the central carbon atom is given in the designations of methylene groups, and hydrogen atoms are indicated without their numbering (example, methylene group C10H_2_). Below, we consider calculations carried out for the cases of DMSO and ethanol solvents, which are used in studying the sample by NMR spectroscopy and UV-Vis electron absorption spectroscopy, respectively.

### 2.1. Theoretically Predicted Structure

In our study we have started with the conformational state that was determined in [3] for the crystalline form. The cytisine moiety in this state has chair conformation, which is more stable for the cytisine molecule according to the Ref. [14]. The conformational state of the complex under study was called state 1, shown in Figure 1a. Its geometry was optimized without any constraints, taking into account the influence of the solution medium. The selected structural parameters of the resulting geometry are given in Table S2.

This conformer is characterized by the formation of a weak interactive hydrogen contact O4H11 (2.276 (2.277) Å length in DMSO (ethanol) solution) with the hydrogen atom in the equatorial position. In order to clarify this interaction, the natural bonding orbitals analysis was performed. The selected results are demonstrated in Table 1. According to it, the occupancies at the H11 and H7 hydrogens in cases of conformers 1 and 2 are slightly raised in C10H11 (conformer 1) and C5H7 (conformer 2) when the O4 oxygen atom is nearby. The opposite situation is observed with C10H12 (conformer 1) and C5H6 (conformer 2). The C10-H11, C10-H12, C5-H6, and C5-H7 bond lengths in both conformers are nearly the same. The LP orbital occupation is also the same.

Thus, the abovementioned moments are in contrary with typical simultaneous peculiarities for the hydrogen bonds:

(1) Growth of C-H length in which H takes part in hydrogen bonds;

(2) Growth of the BD* occupancy;

(3) Decrease in the BD and LP occupancies.

On this basis it was decided that the O4H11 is weak interactive hydrogen contact.

The O4H11 contact is shorter than the contact with the hydrogen atom in the axial position (O4H12, 3.414 (3.415) Å length in DMSO (ethanol) solution) for the closest methylene group (C10H_2_) in the cytisine part. In this case, there is also a contact between the oxygen O8 of the coumarin part and the hydrogen atoms of the opposite methylene group C5H_2_. The contact with the equatorial hydrogen atom H7 (2.902 (2.887) Å length in DMSO (ethanol) solution) is shorter than the contact with the atom H6 (2.996 (2.980) Å length in DMSO (ethanol) solution). It should also be noted that there is a contact between the hydrogen atom of the coumarin part of H34 and the oxygen atom of the intermediate part O4. As in the case of the crystal, in the cytisine part of the molecule there is a shorter hydrogen contact with the equatorial hydrogen atom O1H17 (2.423 Å length in DMSO and ethanol solutions) and a longer hydrogen bond with the axial atom (O1H16, 2.623 (2.622) Å length in DMSO (ethanol) solution). In general, for two solvents (DMSO and ethanol), the approach used predicts the following relationship between the lengths (d) of intramolecular oxygen–hydrogen bonds and contacts:

d(O4H11) < d(O1H17) < d(O1H16) < d(O8H7) < d(O4H34) < d(O8H6) < d(O4H12)(1)

As can be seen from Equation (1) and Table S2, the shortest and strongest intramolecular hydrogen contact is precisely O4H11. In general, it is energetically more favorable to form contacts with equatorial hydrogens than with axial ones.

For this conformational state, in the case of both solvents, the calculation predicts the value of the dihedral angle between the C=O bonds of the cytisine and intermediate parts (O1C19C45O4) to be −11.85°. While between similar bonds in the intermediate and coumarin parts, the value of the dihedral angle O4C45C31O8 is about 97–98°.

Rotation around the C45N9 bond leads to another stable conformational state 2. The calculated electron energy of this state is very close to the energy of conformational state 1 (see Table 2).

State 2 is predicted to be more stable by only 6.2–8.4 × 10^−5^ Ha (0.04–0.05 kcal/mol) as compared with state 1. Such a small difference allows suggesting the existence of approximately equal numbers of molecules in conformational states 1 and 2 at room temperature. State 2 is characterized by the values of the dihedral angles O1C19C45O4 and O4C45C31O8 of about 131.8° and −96–−98°. This corresponds to the joint rotation of the intermediate and coumarin parts relative to the cytisine part. In state 2, similar to state 1, the shortest contact is the O4H7 (2.275 (2.274) Å length in DMSO (ethanol) solution) with the equatorial hydrogen. At the same time, for state 2, the lengths of the O8H11 and O8H12 contacts became close to each other, and the length of the O4H34 contact was in the range of 2.92–2.95 Å. By analogy with Equation (1), for state 2, the following relationship can be written between the lengths of intramolecular hydrogen bonds and contacts:

d(O4H7) < d(O1H17)< d(O1H16)< d(O8H11) < d(O8H12) ≤ d(O4H34) < d(O4H6)(2)

When comparing the optimized geometries for the coumarin and cytisine moieties, a difference in the bond lengths in ring 3 was noted. This is due to the fact that in this ring, the carbon in the CN bond located on the same side as the O= in the intermediate part is more electronegative compared to the carbon on the opposite side. As a result, the CN bond is lengthened and the nearby CC bond is shortened. Compared to state 1, in state 2, the C5N9 bond is lengthened and the C2C5 bond is shortened. On the opposite side of state 2, the situation is different: the C13C10 bond is longer and the C10N9 bond is shorter than in state 1 (see Table S2). The formation of an O4H7 is most clearly seen in the values of the C5N9C45 and C10N9C45 planar angles, as well as the C10N9C45O4 dihedral angle (see Table S2). This structural difference between state 2 and state 1 in the third ring of the cytisine moiety is a consequence of the difference in the charges of the atoms in this ring.

Other stable conformational states result from rotation of the coumarin moiety about the C45C32 bond relative to the cytisine and intermediate moieties as a whole. The structures of these conformational states 3 and 4 are shown in Figure 1c and Figure 1d, respectively. Their energy is higher relative to the more stable state 2 by approximately 0.69–1.09 × 10^−3^ Ha (0.43–0.69 kcal/mol) for DMSO and 0.93–1.45 × 10^−3^ Ha (0.58–0.91 kcal/mol) for ethanol solution. Therefore, this approach predicts a significant proportion of molecules to be in these conformational states. For this theoretical approach, Boltzmann statistics estimates that 20–30% are in states 3 and 4 together at room temperature. The increased total electron energy for states 3 and 4 is due to both the lack of contacts with the methylene hydrogens of C5H2 and C10H2B and the closer proximity of the oxygens O8 and O4, which are separated by about 3.2 Å and 3.7 Å for states 3 and 4. The proximity of the oxygen O8 to H17 (the distance between them is about 2.55 Å) predicts state 4 to be more stable than state 3 in both solvents (see Table 2 and Table S2).

### 2.2. Theoretical and Experimental NMR Spectroscopy Analysis

**Figure 2:**
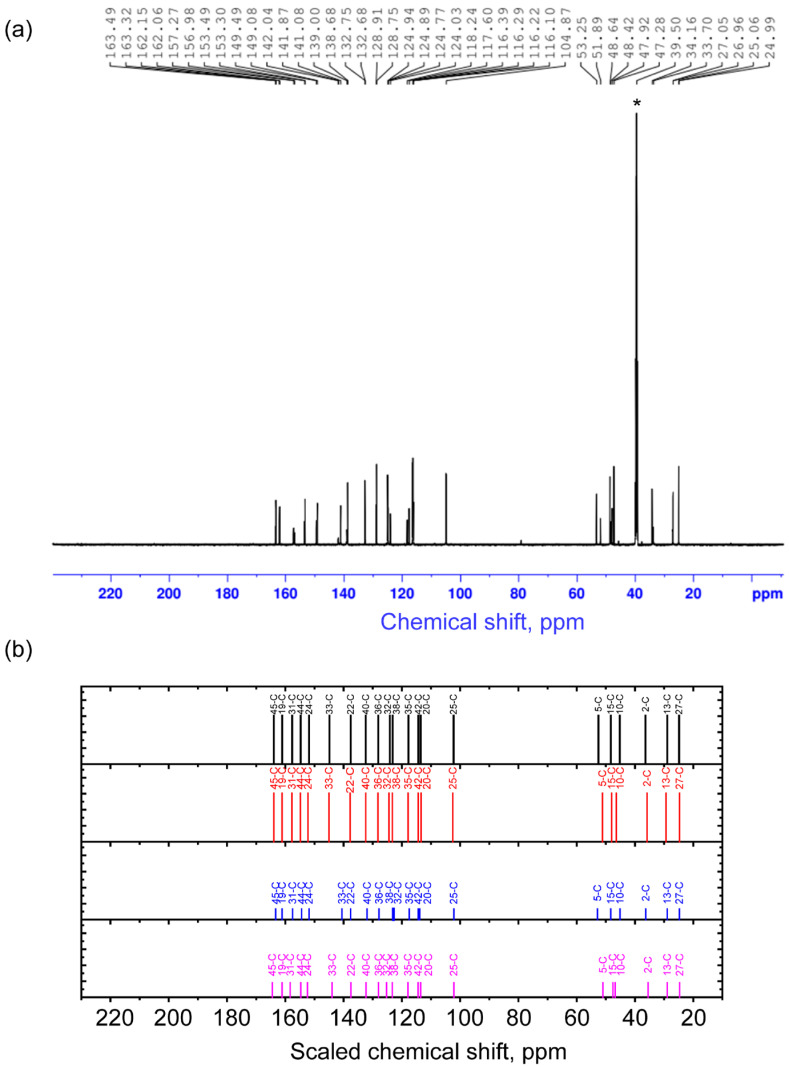
Experimental NMR spectrum (**a**) and scaled calculated chemical shifts (**b**) for the 1st (black), 2nd (red), 3rd (blue), and 4th (purple) conformers on 13C nuclei. The asterisk symbol denotes the residual signal from 13C in non-deuterated solvent molecules (39.5 ppm).

For the established stable conformer states, the magnetic shielding tensors for carbons and protons were calculated using the Gauge-Independent Atomic Orbitals (GIAO) method. From this, chemical shifts were calculated using TMS as a reference. For better agreement between theory and experiment, the scaling procedure was performed. The unscaled and scaled calculated 13C chemical shifts are demonstrated in Table S3 and Table 3 correspondingly. The comparison of the experimental spectrum and the ones predicted for conformers is demonstrated in Figure 2. In the text, further, the scaled values are discussed. Details of calculations are described in Section 3 and Supporting Information (Figure S5) for the four conformers and compared with experimental results.

In the experimental 13C NMR spectrum, the signals are situated in the two spectral regions of chemical shifts. One of them is 24.99–53.25 ppm, and another one is 104.87–163.49 ppm. The range 24.99–53.25 ppm is associated with signals from 13C nuclei in the cytisine moiety, which is confirmed by the proximity of chemical shifts in the spectra of (−)-Cytisine observed in [20]. The range 24.99–53.25 ppm itself is typical for carbon atoms in sp^3^ hybridization [21]. Within this range, carbon atoms bordering other carbons and bonded to one (C2 and C13) and two hydrogens (C27) show smaller chemical shifts compared to carbons (C5, C10, C15) bonded to nitrogen atoms. These results are in agreement with the theory and practical observations in [21]. Calculation of chemical shifts for 13C nuclei in the spectra of different conformers demonstrates that in the range of 24–54 ppm for atoms that are remote from the transition part (C2, C13, C15, C27), the values of chemical shifts depend very weakly on the orientation of the C45O4 bond (dihedral angle O1C19C45O4). At the same time, for C5 and C10 atoms, the calculation predicts significant shifts (see Table 3). Thus, for the C5 atom, the calculated chemical shifts for conformers 1 and 3 are around 52.5–52.9 ppm (experimental value 53.25 ppm), and for conformers 2 and 4—in the range of 46.4–46.85 ppm (experimental value 47.92 ppm). A similar situation occurs for the C10 atom, for which the chemical shifts in conformers 1 and 3 are around 45.2 ppm (experimental value 47.28 ppm), and for conformers 2 and 4—in the range of 50.95–51.2 ppm (experimental value 51.89 ppm). Thus, for the pairs of conformers with the same value as the angle O1C19C45O4, the chemical shifts are close. The observed difference in the chemical shifts in these atoms serves as one of the proofs of the manifestation of two different orientations of the C45O4 bond relative to the cytisine part.

In the experimental 13C NMR spectrum, in the range of 104.87–163.49 ppm, there are signals from carbon atoms forming unsaturated double bonds. According to the experiment and theory, the greatest chemical shift is noted for atoms forming C=O bonds in the cytisine, coumarin, and intermediate parts. A slightly smaller chemical shift is found for signals from atoms C44 and C24 in sp^2^ hybridization. These include C atoms forming bonds with heteroatoms: oxygen and nitrogen, respectively. In the range of 100–148 ppm in the experimental and theoretical spectra, there are signals from carbon atoms in the aromatic part, which have either three bonds with other aromatic carbons or with one hydrogen and two aromatic carbons in the cytisine and coumarin parts.

The presence of various conformers in this spectral region of the experimental spectrum is manifested in the range of 138–142.5 ppm and 116–125 ppm. According to calculations, in the range of 138–142.5 ppm of the experimental spectrum, there is a signal from the nuclei C33 and C22. Moreover, due to the distance from the intermediate region, the signal from the atom C22 is predicted to be approximately the same for all conformers. Along with this, the chemical shift for the nucleus C33 in conformer 3 differs very much from the chemical shifts in conformers 1, 2, and 4, which can be considered its characteristic feature. According to calculations, in the range of 116–125 ppm, there should be a signal from carbon nuclei C32, C38, C35, C42, and C20. Note that nuclei C38, C35, and C42 are in the coumarin part, and C20 is in the cytisine part. They are quite distant from the region where changes occur during binding into a complex. At the same time, the C32 nucleus is on the border of the coumarin and intermediate regions, which makes it sensitive to such changes.

**Table 3:** Interpretation of the 13C NMR spectrum of the complex (the chemical shifts are given scaled with respect to the TMS 13C signal).

Experimental Chemical Shifts for the Complex, ppm	Experimental Chemical Shifts for the Coumarin * and (-)-Cytisine ** Molecules, ppm	Theoretical Chemical Shift, ppm				Assignment
		Conformer 1	Conformer 2	Conformer 3	Conformer 4	
163.49	--	--	--	163.32039	164.4544	45-C
163.32	--	163.93938	163.89789	--	--	45-C
162.15	**163.4**	--	--	161.12527	161.12232	19-C
162.06	**163.4**	161.07417	161.09055	--	--	19-C
157.27	*160.63*	--	--	--	158.30027	31-C
156.98	*160.63*	157.63224	157.70973	157.54976	--	31-C
153.49	*153.99*	154.75016	--	--	--	44-C
153.3	*153.99*	--	154.81441	154.41463	154.69328	44-C
149.49	**150.9**	--	152.20028	--	152.39242	24-C
149.08	**150.9**	151.86926	--	151.87505	--	24-C
141.87	*143.48*	--	--	--	143.92684	33-C
141.08	*143.48*	144.86694	145.03269	--	--	33-C
139	**138.6**	--	--	140.57613	--	33-C
138.68	**138.6**	137.55998	137.76359	137.57685	137.46474	22-C
132.75	*131.79*	132.39204	132.41392	--	132.27445	40-C
132.68	*131.79*	--	--	131.95049	--	40-C
128.91	*127.95*	--	128.19128	--	--	36-C
128.75	*127.95*	128.09928	--	127.86085	127.99149	36-C
124.94	*116.56*	124.14097	124.42785	--	125.25192	32-C
124.89	*116.56*	--	--	--	123.28139	38-C
124.77	*124.43*	123.17321	123.24363	123.14339	--	38-C
124.03	*124.43*	--	--	122.88544	--	32-C
118.24	*118.81*	--	117.89503	--	117.93348	35-C
117.6	*118.81*	117.74929	--	117.46083	--	35-C
116.39	*116.70*	--	114.39789	--	114.45547	42-C
116.29	*116.70*	114.35817	--	114.38426	--	42-C
116.22	**116.4**	113.57598	--	113.87905	113.57324	20-C
116.1	**116.4**	--	113.45574	--	--	20-C
104.87	**104.7**	102.26148	102.52149	102.16693	102.1685	25-C
53.25	**53.8**	52.54109	--	52.86819	--	5-C
51.89	**53.8**	--	51.18896	--	50.97495	10-C
48.64	**52.8**	48.32414	--	48.3112	--	15-C
48.42	**52.8**	--	48.00117	--	47.58756	15-C
47.92	**49.5**	--	46.41982	--	46.84412	5-C
47.28	**49.5**	45.20804	--	45.15351	--	10-C
39.5	--	--	--	--	--	residual 13C in DMSO solvent
34.16	**35.4**	36.39801	--	36.28492	--	2-C
33.7	**35.4**	--	35.84356	--	35.53579	2-C
27.05	**27.5**	--	29.33625	--	--	13-C
26.96	**27.5**	28.94628	--	28.94226	28.94187	13-C
25.06	**26.1**	24.85291	--	24.80348	--	27-C
24.99	**26.1**	--	24.72158	--	24.69314	27-C

* Data taken from the AIST database (SDBS No. 802) [22] is highlighted in bold; ** data taken from [20] is highlighted in italics.

**Figure 3:**
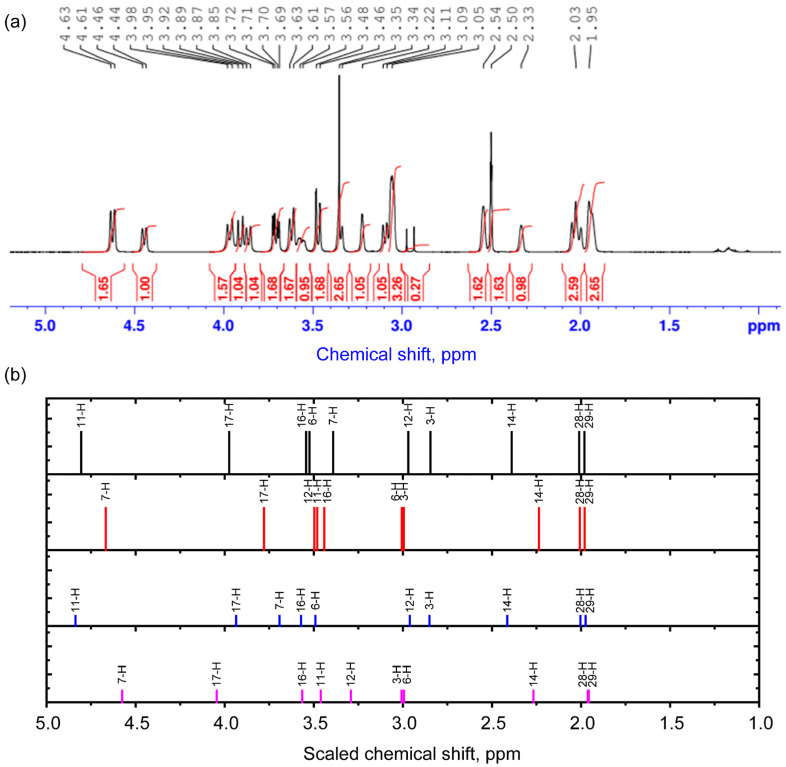
Experimental 1H NMR spectrum in the range of 1–5 ppm (**a**) and scaled calculated chemical shifts (**b**) for the 1st (black), 2nd (red), 3rd (blue), and 4th (purple) conformers for protons. The signal from 1H in non-deuterated solvent molecules is present at 2.5 ppm.

**Figure 4:**
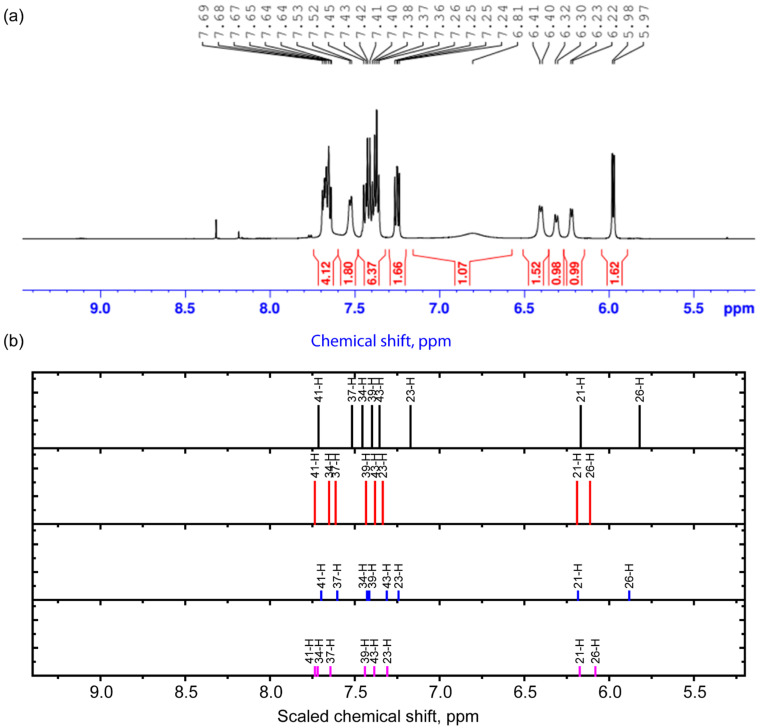
Experimental 1H NMR spectrum in the range 5.2–9.4 ppm (**a**) and scaled calculated chemical shifts (**b**) for the 1st (black), 2nd (red), 3rd (blue), and 4th (purple) conformers for protons in this range.

To characterize the structure of the complex, the 1H NMR spectrum was also studied. The unscaled and scaled theoretical chemical shifts are demonstrated in Table 4 and Table S4. The scaling procedure is demonstrated in Figure S6. The comparison of experimental and theoretical results is demonstrated in Table 4. The spectra obtained in the ranges of 5.2–9.4 ppm and 1–5 ppm are shown in Figure 3 and Figure 4. In the range of 1–5 ppm, there is a signal from the nuclei of H atoms bound to C atoms in sp^3^ hybridization (saturated carbon atoms) according to the theory and Ref. [21]. The calculation predicts the smallest values of chemical shifts for protons that are most distant from oxygen atoms. These include the nuclei of H28, H29, H3, and H14 atoms. The signals of these protons in the experimental spectra of different conformers are located in the range from 1.9 to approximately 3 ppm. At the upper boundary (from about 3 ppm) begins the spectral region where the calculation predicts chemical shifts for protons that form longer (and weaker) hydrogen bonds. These are, for example, hydrogen atoms located in the axial position in ring 3 in cytisine. As the length of the hydrogen bond decreases, the chemical shifts increase, reaching the highest value for proton H11 for conformers 1 and 3 and H7 for conformers 2 and 4. The latter result is a clear confirmation of the presence of conformers with two different dihedral angles, O1C19C45O4. Also sensitive to this angle is the nucleus of the H14 atom, whose chemical shift changes in phase with the chemical shifts in the H11 nucleus in conformers 1 and 3 and the H7 nucleus in conformers 2 and 4. In the range of 3–4 ppm, an overlap of signals from different protons with a weak hydrogen bond or located near it is noted, which, together with the inevitable manifestation of multiplicity, makes it difficult to accurately correlate the peaks.

In the region above 5.9–6.5 ppm in the 1H NMR spectrum, there are peaks corresponding to the signals from the H21 and H26 nuclei. The H21 nucleus is located quite remotely, and therefore its signal is weakly sensitive to conformational changes. This cannot be said about the chemical shifts in the signal from the H26 nucleus—they change out of phase compared to the chemical shifts for the H11 nucleus in conformers 1 and 3 and the H7 nucleus in conformers 2 and 4. This may be due to the fact that when the O4H7 bond is formed, the C5 and C2 carbon atoms become more electronegative, and the C24 atom becomes more electropositive. The observed set of peaks can also confirm different conformational compositions. In the region of 7.37–7.69 ppm, the calculation predicts signals from the nuclei related to the coumarin part (H41, H37, H34, H39, and H43), which are close to those observed in Ref. [22]. The H23 atom exhibits the greatest chemical shift among the nuclei of the cytisine part.

Calculation of the areas of proton peaks in the experimental spectrum and analysis of their ratios, supplemented by interpretation of theoretical results, show that often the ratio of the sum of the contributions of the areas of conformer 1 (A1) and conformer 3 (A3) to the sum of the contributions of conformer 2 (A2) and conformer 4 (A4) can be expressed as (A1 + A3)/(A2 + A4) = 1.6/1. Theoretical estimations on the basis of Table 2 data using Boltzmann population (see details in Section 3.4) predict (A1 + A3)/(A2 + A4) = 0.9/1. Discrepancies between theory and experiment may be due to errors in energy calculations. To improve the accuracy of predicting conformer concentrations, more accurate theoretical approaches are needed that take into account dispersion correction and the contribution of long-range interactions.

### 2.3. Theoretical and Experimental UV-Vis Absorbance Spectroscopy Analysis

**Figure 5:**
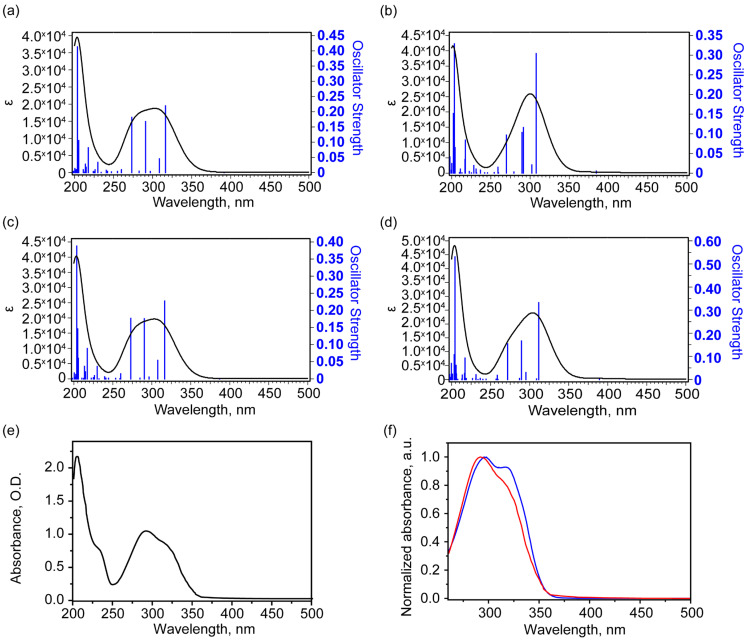
Theoretical electronic UV-Vis absorbance spectra of conformers in ethanol solutions: 1 (**a**), 3 (**b**), 2 (**c**), 4 (**d**), and experimental UV-Vis absorbance spectrum (**e**) and normalized absorbance spectra of ethanol solution (red) and DMSO solution with subtracted solvent contribution (blue) (**f**). In (**f**), the region was reduced due to the strong absorbance of DMSO solvent.

Our study involved modeling 40 lower vertical singlet–singlet electron transitions of the complex in ethanol solutions. The calculated UV-visible absorption spectra for four conformers (Figure 5a–d) and the experimental spectrum (Figure 5e) are presented below. The experimental spectrum is dominated by the absorption peak with a wavelength of 206 nm, against which a shoulder with a wavelength of about 233 nm is visible. A broad band is present in the range of 250–350 nm. It consists of several peaks. The maximum of this band is 292 nm, and a poorly resolved shoulder is noted at about 319 nm. For all four conformers, the calculation predicts the most intense absorption peak at about 204 nm, corresponding to the transition to the excited state of 26 (for conformers 1, 2, and 4) and 25 (for conformer 3). In addition, one transition with a significantly higher oscillator strength is noted at about 217 nm, which may correspond to the 233 nm shoulder in the experimental spectrum. In the range of 250–350 nm, the calculation predicts several electronic transitions (see Table 5) with comparable oscillator strengths. Moreover, for the more stable conformer states 1 and 2, the oscillator strength of the longest wavelength transitions that form this broad band is close to the oscillator strengths of other transitions. And this is more consistent with the observed experimental contour. In contrast, for the higher-energy conformers 3 and 4, the oscillator strength of the transition to the second excited state is 2–3 times greater than the oscillator strengths of other significant transitions that form the band. Figure 5f shows a comparison of normalized UV-visible absorption spectra for a complex-shaped band in the 260–350 nm range from the cytisine–coumarin complex for ethanol and DMSO solutions (in the case of the DMSO solution, the solvent contribution was subtracted). It is in this region that theory predicts a significant difference in absorption for the two groups of conformers: 1, 2 and 3, 4. As can be seen, the normalized spectra in Figure 5f have fairly similar band contour shapes. However, slightly higher absorption is observed at longer wavelengths (approximately 318 nm), specifically for the DMSO sample. This may be due to the slightly higher concentration of conformers 3 and 4 in DMSO. All these facts show that the conformers 3 and 4 exhibit the structure-related properties, which differ from the nearly equally populated lowest states 1 and 2. The redistribution of 1–4 states populations makes the substance promising for optoelectronic applications.

Modeling of UV-visible absorption spectra shows that the dependence of electronic properties, in particular the oscillator strength of singlet–singlet transitions, on the geometry of the molecule is manifested. This property can hypothetically be useful for practical application in optoelectronic devices and systems.

## 3. Materials and Methods

### 3.1. Materials

The current study is a continuation of the investigation presented earlier in the article [3]. In this study, N-(2-oxo-2H-chromen-3-carbonyl)cytisine synthesized in [3] was used to prepare solutions. That work provides a detailed description of the method of its synthesis.

### 3.2. NMR Spectroscopy

The sample was dissolved in 0.52 mL DMSO-d6 (Hexadeuterodimethyl sulfoxide, CAS No. 2206-27-1, 99.9% D, supplied by Merck, Darmstadt, Germany). The solution was placed in a standard 5 mm NMR ampoule. NMR spectra were recorded using a Bruker AV-600 pulsed NMR spectrometer, Bruker, Billerica, MA, USA (resonance frequencies 1H, 13C: 600.18, 150.92 MHz, respectively), equipped with a Z-gradient pulsed field sensor.

The 1H NMR chemical shifts are given relative to the proton signal in tetramethylsilane (TMS). The 13C NMR chemical shifts are given relative to the TMS carbon line. The 1H NMR spectrum was recorded using a 30-degree excitation pulse and a relaxation delay of 5 s. The 13C{1H} NMR spectrum was recorded with broadband decoupling (pulse program—zgpg) and with spectrum editing by signal multiplicity using the J-modulated spin echo method (pulse program—jmod).

### 3.3. UV-Vis Absorbance Spectroscopy

In order to study electronic absorption properties, ethanol and DMSO-d6 solutions with concentrations of 10^−3^ M were prepared. Spectrophotometric grade ethanol was used for solution preparation (CAS No. 64-17-5, purity ≥ 95%, supplier Merck, Darmstadt, Germany) and DMSO-d6 (Hexadeuterodimethyl sulfoxide, CAS No. 2206-27-1, 99.9% D, supplied by Merck). The experimental UV-Vis absorbance spectra were obtained at a spectrophotometer Cary-300 (Agilent Technologies, Santa Clara, CA, USA) in the range of 200–500 nm with a spectral step of 2 nm. Before the measurements, the white light (100%) and dark noise (0%) calibrations were performed. The measurements were performed in the transmission mode and converted into the absorbance (A) optical density units using the expression A = log(1/T), where T is the transmission coefficient.

### 3.4. Theoretical Approach

In the study, the calculations were performed within the density functional theory (DFT) approach using Gaussian G09W Rev. C.01 (Gaussian Inc, Wallingford, CT, USA) software [24]. It used the exchange correlation functional B3LYP [11] and 6-311G(2d,p) basis set [25]. In a number of previous works, this approach demonstrated good predictive ability in modeling electronic [26,27], vibrational [26,27,28,29], and NMR properties [30]. The optimization was performed until the standard conditions for maximum and RMS values of displacements and forces were fulfilled. The identified geometries corresponded to the local minimum point at the total potential energy surface. It was checked during vibrational frequency calculations and found the absence of imaginary modes. The solvent effect was implicitly taken into account within the model of a polarizable medium with standard parameters [31,32,33]. The determined geometries were further used for calculations of the 40 lowest vertical singlet–singlet transitions within the time-dependent DFT calculations. The modeling of the electron absorption spectrum was performed with the full width at half maximum (FWHM) broadening of 0.25 eV for each transition. The DFT calculations were preceded by a search for conformational states using the molecular dynamics method using Forcite software of the Material Studio package. For test purposes, it was used various force fields. Among them were Dreiding, Universal, Compas, CompasII, CompasIII, and FinnisSinclaire force fields [34]. Other parameters were the same. The quality of the geometry search was with the ultrafine preset. The Hessian calculation was included. The charges were defined according to the QEq (Charge Equilibration) method. Among the approaches, the closer geometry to the DFT geometry demonstrated the approach with the Dreiding force field. Further, it was used to calculate conformers energies with variation in the following dihedral angles (the dihedral angle step was 3 degrees, and the number of steps is 120 items per angle): C24C2C5N9, C5C2C24N18, C5C2C27C13, C2C5N9C10, C5N9C10C13, C5N9C45O4, N9C10C13C15, C10C13C15N18, C10C13C27C2, C15N18C24C2, and C31C32C45O4 (the notations correspond to that at Figure 1). This dihedral angle scan was performed with dihedral angles made up of single bonds that are present in the intermediate part, rings 2 and 3 of the cytisine moieties (see Figure 1e for notations).

In order to study the NMR properties of the conformers and their relations with their structures, calculations of the magnetic shielding tensor elements within the Gauge-Independent Atomic Orbital (GIAO) approach [35,36] were performed with the same exchange correlation functional, basis set, and solvent model for the case of DMSO solutions. Also, with the same approach, calculations were performed for the TMS case, which was used as the reference. The isotropic value of the magnetic shielding tensor for the proton and carbon atoms of the conformers (I_conformer_) and TMS (I_ref_) were calculated according to the I_conformer_ = $\frac{\sum_{i=1}^{3}I_{ii,conformer}}{3}$ and I_ref_ = $\frac{\sum_{i=1}^{3}I_{ii,ref}}{3}$ expressions where I_ij,conformer_ (where *i, j* = 1…3) and I_ij,ref_ (where *i, j* = 1…3) are the diagonal components of the magnetic shielding tensor for conformer and TMS reference, respectively. For the TMS reference, the calculated isotropic values of the magnetic shielding tensor in the case of proton and carbon atoms were 184.32 and 31.96 ppm, respectively. The chemical shift for each proton and carbon atom was estimated according to the expression [37]:

Ch. Shift = I_ref_ − I_conformer_.

For greater clarity, when comparing the experimental and calculated data, a procedure of linear scaling of the calculated values of chemical shifts was carried out. The scaled chemical shift (ω_sc_) is related to the originally calculated (ω_theor_) by the relationship ω_sc_ = *a* ω_theor_ + *b*, where *a, b* are the parameters determined by the least squares method when comparing the original theoretical and experimental data. Estimation of the probability of the conformers was performed within the Boltzmann population approach [38,39]. The estimation of p_r_ Boltzmann probability of the r th state was performed in similar way as in [30] according to the $p_{r}=\frac{e^{-\frac{E_{r}}{kT}}}{Z}$, with canonical partition function Z = $\sum_{t=1}^{M}e^{-\frac{E_{t}}{kT}}$, k—Boltzmann constant, E_r_ and E_t_—energy of r th and t th state and T = 298.15 K.

## 4. Conclusions

The study of conformational states of the complex molecule N-(2-oxo-2H-chromene-3-carbonyl)cytisine in solutions was performed. Within the framework of quantum chemical calculations at the B3LYP/6-311G(2d,p) level, the presence of four stable states was predicted. Two of them (conformer 1 and conformer 2) are associated with rotation around the N-C bond at the boundary of the cytisine and intermediate fragments. In these conformers, such rotation is accompanied by the formation of a relatively short contact between the oxygen atom O(=C) of the intermediate moiety and the hydrogen atom H(-C) of the cytisine moiety. The calculation predicts approximately equal concentrations of these conformers. The other two conformers (conformers 3 and 4) are associated with the rotation of the coumarin part at the C-C bond boundary of the coumarin and intermediate moieties. The energies of these conformers are predicted to be higher by at least 0.6 kcal/mol, which makes their expected concentration lower. Theoretical modeling of chemical shifts for the molecule in solution was performed. Spectral features characteristic of different conformers were identified in the experimentally observed 1H and 13C NMR spectra in DMSO-d6. Thus, in the experimental NMR spectrum, the signals from 13 C nuclei are located in the ranges of 47.2–53.3 and 116–25 ppm, and from 1H nuclei in the ranges of 4.4–4.7 and 5.97–6.41 ppm. Based on the 13C and 1H NMR spectra, the presence of two groups of conformers (conformers 1 and 3, as well as conformers 2 and 4) was confirmed. Using this interpretation, it was determined from the ratio of proton areas that the concentration ratio of these groups of conformers is approximately 1.6/1. In addition, a theoretical and experimental study of the optical properties of the complex molecule in ethanol solutions was carried out using UV-Vis spectroscopy in the range of 200–500 nm. Modeling of the electron absorption spectra also confirms that these spectra are formed by an ensemble of conformer states, to which the main contributions are presumably made by conformers 1 and 2, and to a lesser extent by conformers 3 and 4. This study of structural states revealed that, in addition to the structure previously established in the crystalline state, three additional conformational states with relatively low energy exist in solution. The theoretically calculated energy increase for these conformers is no more than 0.69 and 0.91 kcal/mol in DMSO and ethanol solvents, respectively. The information obtained demonstrates the importance of studying the structure of complex molecules consisting of coumarin and cytisine parts connected by a flexible intermediate part. The results obtained in this work may be useful both in studying the binding processes of this molecule in biological systems, such as proteins, and in studying structure-dependent electronic properties important for optoelectronic applications, such as molecular sensors.

## Figures and Tables

**Table 1:** Selected bonds NBO analysis for conformers 1 and 2 **.

NBO	Occupancy	
	Conformer 1	Conformer 2
BD (1) C10–H11	1.97983	1.97658
BD (1) C10–H12	1.97714	1.97838
BD* (1) C10–H11	0.01446	0.01166
BD* (1) C10–H12	0.02008	0.02318
BD (1) C5–H6	1.97679	1.97558
BD (1) C5–H7	1.97707	1.98012
BD* (1) C5–H6	0.02349	0.02064
BD* (1) C5–H7	0.01115	0.01409
LP (1) O4	1.97772	1.97773
LP (2) O4	1.86858	1.86862

** Hereafter, it is used in the following notations: BD—bonding orbital, BD*—antibonding orbital, LP—lone pair.

**Table 2:** Total potential energy of conformers for DMSO and ethanol solutions.

Solvent	Conformer Total Potential Energy, Ha (Relative Potential Energy with Respect to Conformer 2, kcal/mol)			
	1	2	3	4
DMSO	−1221.865439 (0.04)	−1221.865501 (0)	−1221.864415 (0.69)	−1221.864816 (0.43)
Ethanol	−1221.864674 (0.05)	−1221.864758 (0)	−1221.863311 (0.91)	−1221.863827 (0.58)

**Table 4:** Interpretation of the 1H NMR spectrum of the complex (the chemical shifts are scaled with respect to the TMS 1H signal).

Experimental Chemical Shifts for the Complex, ppm	Experimental Chemical Shifts for the Coumarin * and (-)-Cytisine ** Molecules, ppm	Theoretical Chemical Shift, ppm				Assignment
		Conformer 1	Conformer 2	Conformer 3	Conformer 4	
7.67–7.69	*7.532*	7.71451	7.73565	7.69828	7.73507	41-H
7.65–7.64	*7.727*	--	7.65159	--	7.71932	34-H
7.53–7.52	*7.498*	--	7.61335	--	7.64381	37-H
7.53–7.52	*7.498*	7.5168	--	7.60374	--	37-H
7.45–7.43	*7.727*	7.45532	--	7.42774	--	34-H
7.42–7.38	*7.285*	7.39892	7.4337	7.41468	7.44042	39-H
7.37–7.36	*7.320*	7.35425	7.38086	7.3116	7.38547	43-H
7.25–7.26	**7.30**	--	7.33484	--	7.3091	23-H
7.25–7.24	**7.30**	7.17133	--	7.24252	--	23-H
6.41–6.4	**6.45**	--	6.18931	6.18421	6.17365	21-H
6.32–6.3	**6.45**	6.1675	--	--	--	21-H
6.23–6.22	**6.00**	--	6.11264	--	6.08142	26-H
5.98–5.97	**6.00**	5.8203	--	5.88198	--	26-H
4.63–4.61	**3.02**	4.80551	--	4.8375	--	11-H
4.46–4.44	**3.08**	--	4.66794	--	4.57591	7-H
3.98–3.85	**4.13**	3.9746	3.7792	3.9355	4.04502	17-H
3.34–3.72	**3.13**	2.96933	3.49704	2.96097	3.29203	12-H
3.34–3.72	**3.89**	3.54296	3.44132	3.57197	3.56477	16-H
3.34–3.72	**3.02**	--	3.48042	--	3.46063	11-H
3.34–3.72	**3.08**	3.39088	--	3.69225	--	7-H
3.34–3.72	**3.02**	3.52404	--	3.49041	--	6-H
3.22–3.05	**2.91**	2.84501	2.99421	2.85068	3.00766	3-H
3.22–3.05	**3.02**	--	3.00641	--	2.99382	6-H
2.54	**2.35**	2.38887	--	2.41395	--	14-H
2.5	--	--	--	--	--	Residual 1H in DMSO-d6 solvent
2.33	**2.35**	--	2.23651	--	2.26686	14-H
2.03	**1.96**	2.01074	2.00632	2.00363	1.95637	28-H
1.95	**1.96**	1.98077	1.97923	1.97443	1.96155	29-H

* Data taken from the AIST database (SDBS No. 802) [22] is highlighted in italics; ** data taken from [23] is highlighted in bold.

**Table 5:** Parameters for the selected singlet–singlet vertical transitions in the 200–500 nm region. The frontier orbitals are demonstrated in Figure S7.

Conformer	Excited State No	Orbitals with > 14% Contribution (Percent)	Oscillator Strength	Wavelength, nm (Energy, eV)
1	2	94 (HOMO-1) -> 96 (LUMO) (79)	0.2191	316.44 (3.9181)
	5	95 (HOMO) -> 97 (LUMO + 1) (94)	0.1676	290.97 (4.2610)
	7	90 (HOMO-5) -> 96 (LUMO) (75)	0.1814	273.25 (4.5374)
	19	95 (HOMO) -> 101(LUMO + 5) (64) 95 (HOMO) -> 100 (LUMO + 4) (21)	0.0816	217.61 (5.6977)
	26	91 (HOMO-4) -> 98 (LUMO + 2) (23) 92 (HOMO-3) -> 98 (LUMO + 2) (19) 93 (HOMO-2) -> 99 (LUMO + 3) (16) 87 (HOMO-8) -> 96 (LUMO) (15)	0.4132	203.71 (6.0862)
2	2	94 (HOMO-1) -> 96 (LUMO) (79)	0.2273	316.43 (3.9183)
	5	95(HOMO) -> 97(LUMO + 1) (94)	0.1756	290.38 (4.2698)
	7	90 (HOMO-5) -> 96 (LUMO)(75)	0.1765	273.05 (4.5407)
	19	95 (HOMO) -> 101 (LUMO + 5) (69) 95 (HOMO) -> 100 (LUMO + 4) (15)	0.0885	217.23 (5.7076)
	26	91(HOMO-4) -> 98 (LUMO + 2) (26) 93 (HOMO-2) -> 99 (LUMO + 3) (19) 87 (HOMO-8) -> 96 (LUMO) (15)	0.3879	203.66 (6.0877)
3	2	94 (HOMO-1) -> 96 (LUMO) (90)	0.3041	307.82 (4.0278)
	4	91(HOMO-4) -> 96 (LUMO) (63) 95(HOMO) -> 97(LUMO + 1) (22)	0.1159	291.46 (4.2539)
	5	95(HOMO) -> 97 (LUMO + 1)(74) 91 (HOMO-4) -> 96 (LUMO) (17)	0.1031	289.59 (4.2813)
	7	90(HOMO-5) -> 96 (LUMO) (70) 88 (HOMO-7) -> 96 (LUMO) (15)	0.0965	269.80 (4.5955)
	20	94 (HOMO-1) -> 99 (LUMO + 3) (41) 94 (HOMO-1) -> 98 (LUMO + 2) (20)	0.0348	216.94 (5.7152)
	25	91(HOMO-4) -> 98 (LUMO + 2) (33) 93(HOMO-2) -> 99 (LUMO + 3) (17)	0.3287	203.47 (6.0934)
4	2	94 (HOMO-1) -> 96 (LUMO) (90)	0.3324	311.68 (3.9779)
	5	95(HOMO) -> 97(LUMO + 1) (96)	0.1665	289.44 (4.2836)
	7	90 (HOMO-5) -> 96 (LUMO) (74)	0.1539	271.78 (4.5619)
	19	95(HOMO) -> 101 (LUMO + 5) (80)	0.0925	217.15 (5.7097)
	26	91 (HOMO-4) -> 98 (LUMO + 2) (41) 94 (HOMO-1) -> 98 (LUMO + 2) (14)	0.5305	204.23 (6.0708)

## Data Availability

The original contributions presented in this study are included in the article and Supplementary Materials. Further inquiries can be directed to the corresponding authors.

## Supplementary Materials

The following supporting information can be downloaded at: https://www.mdpi.com/article/10.3390/molecules30204139/s1, Figure S1: Non-optimized geometry total energy for 6000 lowest energy geometries of conformers (**a**) and corresponding optimized total energies for the same conformers (**b**), Figure S2: The examples of 6 optimized geometries with various energy levels 1 (**a**), 2 (**b**),3 (**c**), 4 (**d**), 5 (**e**), 6 (**f**) are demonstrated, Figure S3: The atomic displacements in the vibrational mode with imaginary frequency (55i cm^−1^), Figure S4: Potential energy scan with C5N9C45C32 dihedral angle change (**a**), and C31C32C45N9 starting from state 1 (**b**) and state 2 (**c**); Table S1: Optimized geometries of conformers in DMSO and ethanol solutions; Table S2. Selected structural peculiarities of various conformers in DMSO and ethanol solutions; Figure S5: The linear regression between theoretical unscaled and experimental 13C NMR chemical shifts for the selected signals. For linear regression, the intercept value is −4.647 ± 0.710, and the slope is 0.981 ± 0.006. The Pearson’s r is 0.999. To construct this dependence, individual values were selected that are interpreted unambiguously and correspond to well-resolved signals; Table S3: Interpretation of the 13C NMR spectrum of complex (the chemical shifts are given unscaled as calculated with respect to the TMS 13C signal); Figure S6: The linear regression between theoretical unscaled and experimental 13C NMR chemical shifts for the selected signals. For linear regression, the intercept value is 0.061 ± 0.003, and the slope is 0.961 ± 0.004. The Pearson’s r is 0.999. To construct this dependence, individual values were selected that are interpreted unambiguously and correspond to well-resolved signals; Table S4: Interpretation of the 1H NMR spectrum of complex (the chemical shifts are given scaled with respect to the TMS 13C signal); Figure S7: HOMO and LUMO orbitals for conformers 1 (**a**), 2 (**b**), 3 (**c**), and 4 (**d**).

## Abbreviations

The following abbreviations are used in this manuscript:

NMR	Nuclear Magnetic Resonance
DMSO	Dimethyl Sulfoxide
NBO	Natural Bonding Orbitals
UV-Vis	Ultraviolet-Visible
AIST	Advanced Industrial Science and Technology
SDBS	Spectral Database for Organic Compounds
CAS	Chemical Abstracts Service
TMS	Tetramethylsilane
DFT	Density Functional Theory
B3LYP	Becke, 3-parameter, Lee–Yang–Parr (exchange correlation functional)
FWHM	Full Width at Half Maximum
GIAO	Gauge-Independent Atomic Orbital
