# Supplementary material for: Investigation of N-(2-oxo-2H-chromen-3-carbonyl)cytisine’s Molecular Structure in Solution
Source: Molecules. 2025 Oct 20;30(20):4139. doi: 10.3390/molecules30204139 (PMC12565994; doi:10.3390/molecules30204139)
Supplement: Supplementary file 1 [file molecules-30-04139-s001.zip › molecules-3883521-supplementary.pdf]

Supporting information

for

Investigation of N-(2-oxo-2H-chromen-3-carbonyl)cytisine's Molecular Structure in solution.

Kymbat Kopbalina [1], Aigerim Adekenova [2], Zhanar Shaimerdenova [2], Zhanargul Kairatova [2], Kuanysh Shakarimova [2], Dmitrii Pankin [3], Mikhail Smirnov [4], Anarkul Kishkentayeva [2,*], Makpal Artykbayeva [5] and Roza Jalmakhanbetova [5,*]

[1] Department of Physics and Nanotechnology, Buketov Karaganda University, Universitetskaya 28, Karaganda 100024, Kazakhstan
[2] School of Pharmacy, Karaganda Medical University, Karaganda 100012, Kazakhstan
[3] Center for Optical and Laser Materials Research, St. Petersburg State University, Ulianovskaya 5, 198504 St. Petersburg, Russia
[4] Faculty of Physics, St. Petersburg State University, Universitetskaya Nab. 7/9, 199034 St. Petersburg, Russia
[5] Department of Chemistry, Faculty of Natural Sciences, L.N. Gumilyov Eurasian National University, Astana 010000, Kazakhstan
* Correspondence: anar_kish@mail.ru (A.K.); rjalmakhanbetova@gmail.com (R.J.)

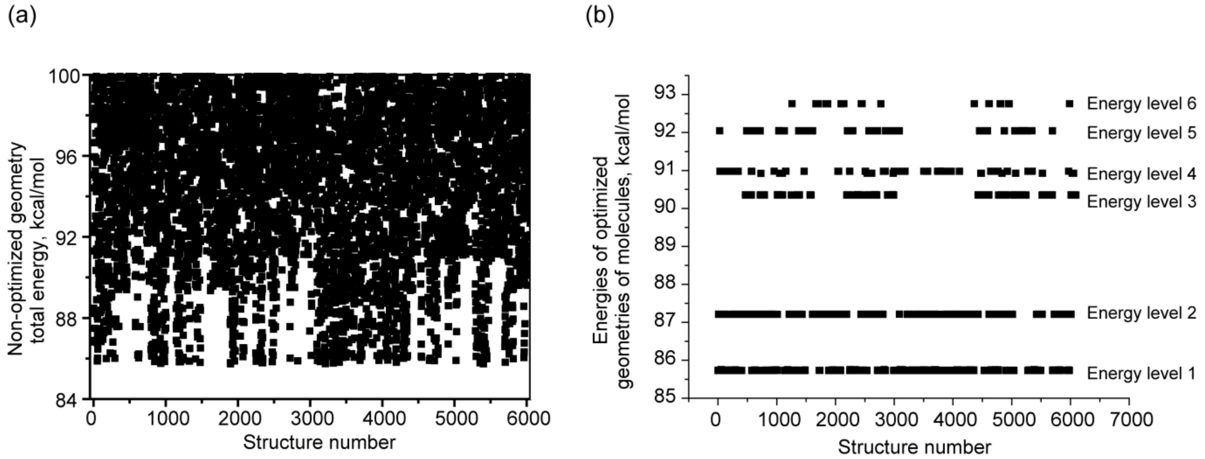

Figure S1. Non-optimized geometry total energy for 6000 lowest energy geometries of conformers (a) and corresponding optimized total energies for the same conformers (b).

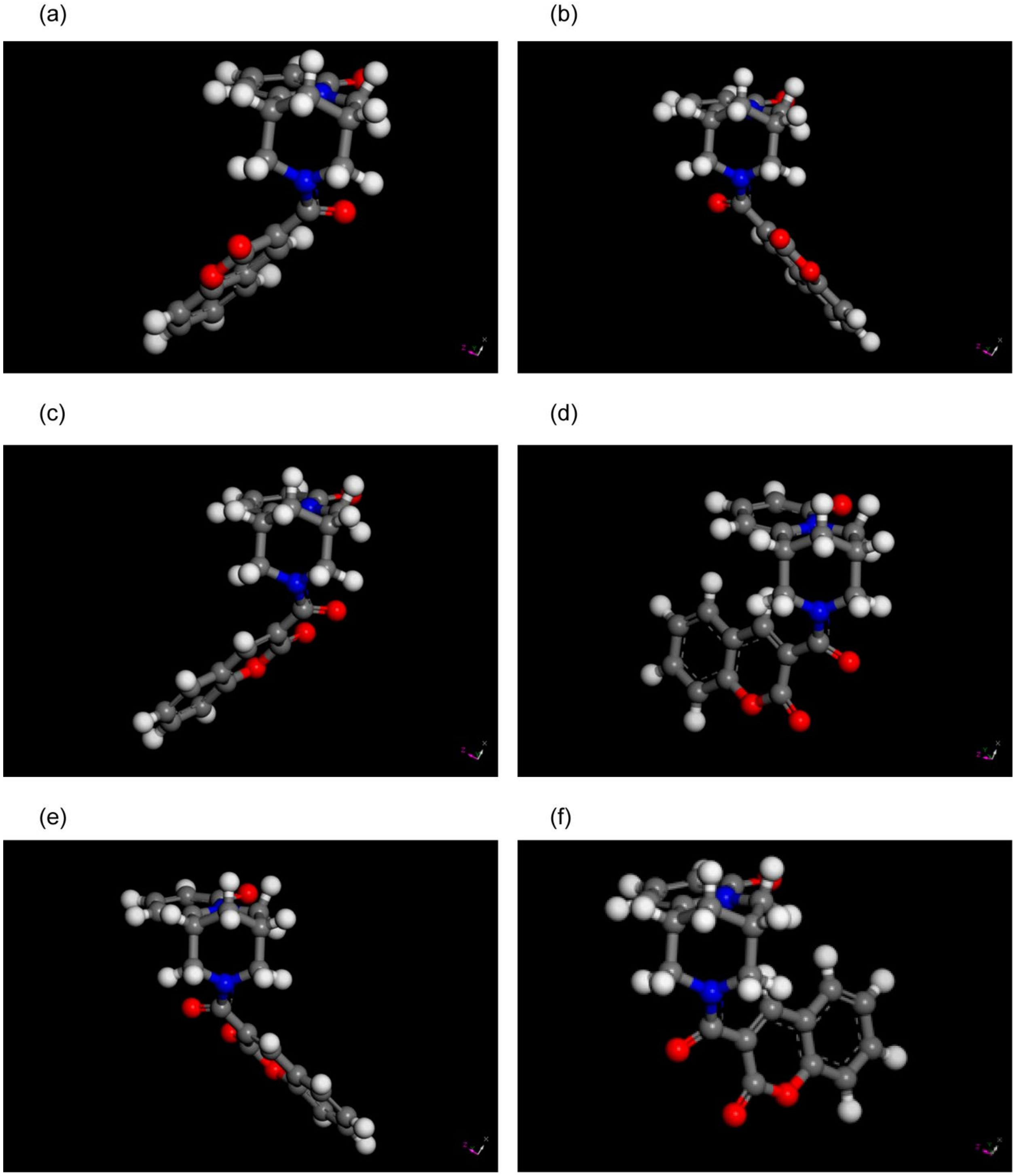

Figure S2. The examples of 6 optimized geometries with various energy levels 1 (a), 2 (b),3 (c), 4 (d), 5 (e), 6 (f) are demonstrated.

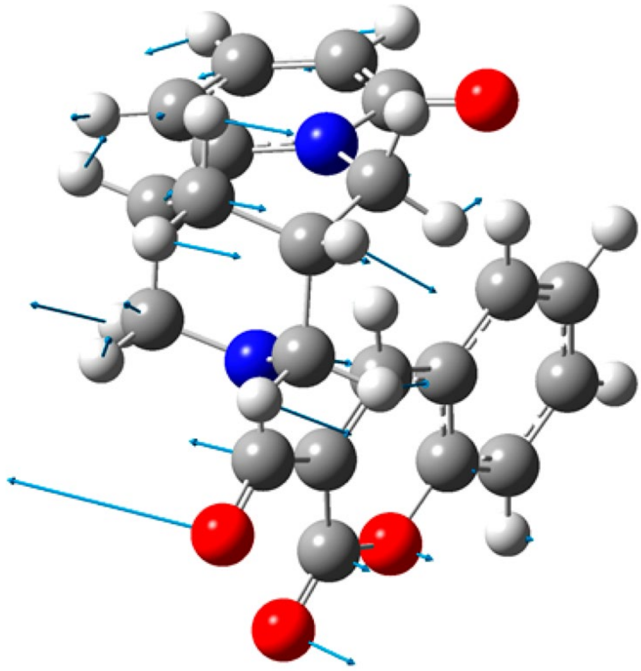

Figure S3. The atomic displacements in the vibrational mode with imaginary frequency (55i cm^{-1}).

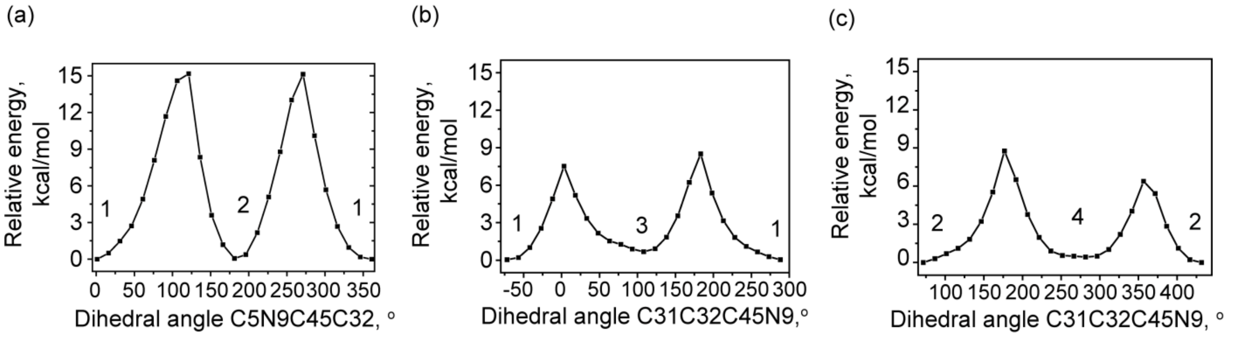

Figure S4. Potential energy scan with C5N9C45C32 dihedral angle change (a), and C31C32C45N9 starting from state 1 (b) and state 2 (c).

The potential energy scan was performed in relaxed regime. It means that all other structural parameters were relaxed expect the changing dihedral angle. The scan step was 15°. All other parameters were as described in section 3.4 in manuscript.

Table S1. Optimized geometries of conformers in DMSO and ethanol solutions.

Conformer 1 in DMSO				Conformer 1 in ethanol			
O	-3.47490700	2.46938600	1.65026100	O	-3.47496700	2.47554700	1.64366600
C	-2.23058000	-0.70426800	-1.69112400	C	-2.23493100	-0.71095400	-1.68670900
H	-2.12986400	-0.65361600	-2.77599500	H	-2.13650500	-0.66468800	-2.77203700

O	-0.21711300	-1.50065400	2.37662500	O	-0.21478200	-1.48683600	2.38037100
C	-1.01380700	-1.50020200	-1.16168500	C	-1.01654700	-1.50405900	-1.15655400
H	-0.99596200	-2.48120900	-1.64753800	H	-0.99825200	-2.48674400	-1.63899800
H	-0.09606500	-0.97892400	-1.41793700	H	-0.09988900	-0.98274400	-1.41656600
O	1.68941600	-3.05060700	-0.44788400	O	1.68197700	-3.04332300	-0.46090200
N	-1.09394100	-1.66800000	0.28490000	N	-1.09363800	-1.66642600	0.29059900
C	-2.36191900	-2.20947600	0.78043200	C	-2.36020200	-2.20658700	0.79077400
H	-2.33163000	-2.17490800	1.86578100	H	-2.32750000	-2.16803400	1.87593700
H	-2.43979000	-3.25846700	0.47196100	H	-2.43821300	-3.25682600	0.48642600
C	-3.56707700	-1.43545500	0.22955500	C	-3.56696600	-1.43518000	0.23967800
H	-4.46200700	-1.95547200	0.57881600	H	-4.46095500	-1.95420200	0.59291500
C	-3.65914500	-0.00366600	0.75823100	C	-3.65844700	-0.00129500	0.76295700
H	-4.69294800	0.34440400	0.71076700	H	-4.69251600	0.34622300	0.71696600
H	-3.36433100	0.05405600	1.80519300	H	-3.36103600	0.06093600	1.80892300
N	-2.84130500	0.98901700	0.02054700	N	-2.84310800	0.98879100	0.01919000
C	-2.84996000	2.28100300	0.60146900	C	-2.85176400	2.28345000	0.59501300
C	-2.11173900	3.28454600	-0.11079100	C	-2.11515000	3.28446900	-0.12291000
H	-2.09776000	4.27282700	0.32841100	H	-2.10101300	4.27447600	0.31235800
C	-1.48639700	3.00230400	-1.28774700	C	-1.49210500	2.99776100	-1.29986700
H	-0.94736700	3.78013100	-1.81672300	H	-0.95464100	3.77375300	-1.83316300
C	-2.20910300	0.71241200	-1.16365300	C	-2.21325200	0.70789100	-1.16513900
C	-1.53688300	1.70178100	-1.82952700	C	-1.54299500	1.69492800	-1.83638900
H	-1.04641900	1.46697500	-2.76377800	H	-1.05459100	1.45667700	-2.77087400
C	-3.52519700	-1.41809200	-1.29874300	C	-3.52843900	-1.42379400	-1.28879000
H	-3.52934300	-2.43423600	-1.69963100	H	-3.53306100	-2.44153900	-1.68568000
H	-4.39408400	-0.89788300	-1.70870000	H	-4.39842400	-0.90552900	-1.69896700
O	3.30151000	-1.53574500	-0.53359700	O	3.29829300	-1.53264800	-0.54251100
C	2.03781900	-1.93278400	-0.14485900	C	2.03414600	-1.92846400	-0.15245000
C	1.24578800	-0.96686800	0.60671800	C	1.24716900	-0.96405200	0.60676600
C	1.76302800	0.23944000	0.93076200	C	1.76937000	0.23873300	0.93606100
H	1.18186900	0.93567400	1.52509400	H	1.19217500	0.93266600	1.53689200
C	3.07437000	0.62539600	0.50419700	C	3.08045800	0.62333800	0.50765600
C	3.65416500	1.87351500	0.78575000	C	3.66478200	1.86815500	0.79440300
H	3.08659100	2.59823200	1.35690600	H	3.10099300	2.59134600	1.37125100
C	4.92816200	2.16817300	0.33974700	C	4.93839500	2.16147600	0.34649400
H	5.37108700	3.13100900	0.55915800	H	5.38485400	3.12174100	0.57001300
C	5.64789400	1.22017500	-0.39647000	C	5.65323900	1.21531500	-0.39679800
H	6.64644800	1.45327900	-0.74443400	H	6.65160200	1.44726000	-0.74612400
C	5.09900000	-0.01898900	-0.68777200	C	5.09984500	-0.02057100	-0.69330300
H	5.64178200	-0.76388000	-1.25474900	H	5.63881500	-0.76414800	-1.26561500
C	3.81714100	-0.30444000	-0.23508800	C	3.81832600	-0.30475400	-0.23871800
C	-0.08968200	-1.40519400	1.15827100	C	-0.08806700	-1.39899000	1.16163100
Conformer 2 in DMSO				Conformer 2 in ethanol			
O	-2.03633700	2.96436100	1.36165000	O	-2.08273900	2.96336800	1.36671100
C	-3.34822900	-1.40666100	-0.03700600	C	-3.34042200	-1.41971600	-0.04240900
H	-4.27160300	-1.82347700	-0.44113100	H	-4.25841400	-1.84617100	-0.44879200
O	-0.18774000	-0.89541400	-2.40603300	O	-0.17782200	-0.88251300	-2.40396400
C	-2.18309800	-2.00634000	-0.85314600	C	-2.16726600	-2.00700300	-0.85545500
H	-2.23291300	-3.09890100	-0.78175100	H	-2.20674300	-3.10017700	-0.78562900
H	-2.24625700	-1.72412900	-1.89948500	H	-2.22972600	-1.72416700	-1.90170000
O	1.96546800	-2.97043600	-0.16345400	O	1.96842300	-2.96048500	-0.15621600
N	-0.89117200	-1.54970700	-0.34396000	N	-0.88084500	-1.53935200	-0.34259000
C	-0.68995200	-1.72083300	1.09445900	C	-0.68142900	-1.70824400	1.09618200

H	0.22840100	-1.21952300	1.38960300	H	0.23085500	-1.19685500	1.39348800
H	-0.57815000	-2.78615300	1.32111600	H	-0.55847200	-2.77218200	1.32354200
C	-1.86908500	-1.15210700	1.90082800	C	-1.86857200	-1.15131300	1.89909400
H	-1.67842400	-1.40080400	2.94726300	H	-1.67857700	-1.39839500	2.94606600
C	-1.97173300	0.37137500	1.82501800	C	-1.98661500	0.37116400	1.82341800
H	-2.49192300	0.75161500	2.70659000	H	-2.51010600	0.74594400	2.70537800
H	-0.98754100	0.83823900	1.82628000	H	-1.00732800	0.84847600	1.82467500
N	-2.69832800	0.89221100	0.64267100	N	-2.71896600	0.88553100	0.64183700
C	-2.67070400	2.30407100	0.53178200	C	-2.70781600	2.29809600	0.53423500
C	-3.41238100	2.85265100	-0.56687200	C	-3.45378400	2.84003600	-0.56500500
H	-3.39076500	3.92895100	-0.67130500	H	-3.44500000	3.91674800	-0.66689400
C	-4.11910300	2.05294000	-1.41367900	C	-4.14772600	2.03335000	-1.41561200
H	-4.68427400	2.49062100	-2.22880000	H	-4.71594100	2.46574600	-2.23146300
C	-3.40816600	0.09339500	-0.21588100	C	-3.41667200	0.07985200	-0.22009500
C	-4.12474700	0.65435200	-1.23850400	C	-4.13704600	0.63432800	-1.24344600
H	-4.68058000	0.01077600	-1.90572500	H	-4.68380400	-0.01417400	-1.91340200
C	-3.18369400	-1.77962200	1.43738600	C	-3.17582400	-1.79130300	1.43218200
H	-3.15791000	-2.86611200	1.54861300	H	-3.14025600	-2.87754700	1.54335300
H	-4.02282100	-1.40750900	2.03002600	H	-4.01973100	-1.42686200	2.02281300
O	3.54809100	-1.45741200	0.16330300	O	3.55729500	-1.45240100	0.16363000
C	2.26815300	-1.80093600	-0.22197800	C	2.27514500	-1.79247400	-0.21839100
C	1.40331000	-0.71844400	-0.67484800	C	1.41374600	-0.70770300	-0.67334000
C	1.87251100	0.54707700	-0.75254700	C	1.88697800	0.55614800	-0.75311100
H	1.23475300	1.33867600	-1.12980500	H	1.25191600	1.34904100	-1.13229000
C	3.20507700	0.87242600	-0.34154500	C	3.22041400	0.87818900	-0.34203600
C	3.73909500	2.17126800	-0.37255700	C	3.75772800	2.17572100	-0.37234100
H	3.11818000	2.98509300	-0.72727000	H	3.13895500	2.99135400	-0.72670000
C	5.03595700	2.40287900	0.04379600	C	5.05490600	2.40387000	0.04477100
H	5.44361300	3.40514900	0.01835100	H	5.46501400	3.40518000	0.02027700
C	5.82432900	1.34006700	0.49954000	C	5.84061700	1.33884700	0.49992300
H	6.84038600	1.52431500	0.82544500	H	6.85712000	1.52044400	0.82598200
C	5.32184500	0.04870400	0.53941100	C	5.33501800	0.04871200	0.53881200
H	5.91803900	-0.78453300	0.88747900	H	5.92900900	-0.78638700	0.88620600
C	4.01680000	-0.17293600	0.11834100	C	4.02939100	-0.16940000	0.11783600
C	0.03555400	-1.06809500	-1.21036300	C	0.04566900	-1.05566300	-1.20869800
Conformer 3 in DMSO				Conformer 3 in ethanol			
O	4.02393200	1.85772000	-1.52228900	O	4.03033800	1.84988300	-1.53205700
C	2.02111100	-0.92487900	1.79649300	C	2.02312700	-0.91388700	1.79920200
H	1.87510500	-0.82597200	2.87287400	H	1.87867200	-0.80906800	2.87529700
O	0.06999500	-1.54179700	-2.35894000	O	0.05597100	-1.57536700	-2.34708700
C	0.70539800	-1.49495300	1.21736900	C	0.70668900	-1.48711000	1.22515200
H	0.48456900	-2.43869000	1.72889000	H	0.48559600	-2.42694400	1.74408600
H	-0.11536000	-0.81009000	1.40698600	H	-0.11358800	-0.80039200	1.41061200
O	-0.50607800	1.47939700	-1.45629700	O	-0.49742400	1.46420000	-1.44944800
N	0.82161600	-1.73409800	-0.21920000	N	0.82073100	-1.73679400	-0.20955300
C	1.99314900	-2.51427700	-0.62895500	C	1.99193300	-2.51865100	-0.61645200
H	2.02434400	-2.51262900	-1.71492300	H	2.02100800	-2.52348400	-1.70249400
H	1.86319600	-3.54901600	-0.29199400	H	1.86371200	-3.55173500	-0.27335700
C	3.29006200	-1.95292200	-0.03090000	C	3.28942100	-1.95264200	-0.02403500
H	4.09039100	-2.63810800	-0.31931000	H	4.08966200	-2.63921800	-0.30946800
C	3.66868200	-0.57943400	-0.58881100	C	3.66644400	-0.58221000	-0.59097900
H	4.74771300	-0.43210600	-0.51209600	H	4.74592000	-0.43523800	-0.52042600
H	3.42359200	-0.49856900	-1.64722700	H	3.41653800	-0.50679500	-1.64872900

N	3.03415200	0.57283600	0.09505600	N	3.03622100	0.57404200	0.08955700
C	3.32873900	1.82304800	-0.50182800	C	3.33390300	1.82132100	-0.51281900
C	2.78078900	2.97059600	0.16301700	C	2.78731500	2.97275000	0.14680600
H	2.98766800	3.93131600	-0.28865200	H	2.99540600	3.93090600	-0.30965300
C	2.05483100	2.84595000	1.30863600	C	2.06106400	2.85451200	1.29270200
H	1.66081100	3.72786100	1.80085100	H	1.66805900	3.73929700	1.78058100
C	2.30118500	0.45507500	1.24670200	C	2.30368100	0.46299900	1.24189100
C	1.81256600	1.57381600	1.86605600	C	1.81721800	1.58526000	1.85636800
H	1.24098300	1.46369000	2.77685100	H	1.24612600	1.48019900	2.76810100
C	3.17680300	-1.87975300	1.49187700	C	3.17834600	-1.87065600	1.49848000
H	2.97038200	-2.86600600	1.91405200	H	2.97254800	-2.85455600	1.92656800
H	4.10598500	-1.51780600	1.93815600	H	4.10812200	-1.50617700	1.94148500
O	-2.59749900	1.43392500	-0.73265300	O	-2.59164500	1.43067600	-0.73168400
C	-1.40000500	0.80375700	-1.00486400	C	-1.39603500	0.79418400	-1.00005700
C	-1.32441500	-0.62213300	-0.70459300	C	-1.32884200	-0.63202300	-0.69826200
C	-2.38711400	-1.27365500	-0.18427600	C	-2.39605500	-1.27760900	-0.18023700
H	-2.32732500	-2.33460700	0.03404600	H	-2.34240600	-2.33884800	0.03849400
C	-3.61837900	-0.58941500	0.08413900	C	-3.62450700	-0.58672000	0.08478000
C	-4.75753400	-1.21014200	0.62157300	C	-4.76811300	-1.20096900	0.62007900
H	-4.71813900	-2.26768300	0.85388100	H	-4.73481500	-2.25849400	0.85354800
C	-5.90894300	-0.48053500	0.84923400	C	-5.91646700	-0.46542000	0.84405900
H	-6.78446600	-0.96342600	1.26370200	H	-6.79546500	-0.94352500	1.25674700
C	-5.94347700	0.88439800	0.54323100	C	-5.94340500	0.89925200	0.53633200
H	-6.84694300	1.45400800	0.72174300	H	-6.84449500	1.47358100	0.71174400
C	-4.83223700	1.52115500	0.01241500	C	-4.82765600	1.52971400	0.00757700
H	-4.84103800	2.57583600	-0.22954900	H	-4.83046400	2.58408500	-0.23588200
C	-3.67992500	0.77840100	-0.21146300	C	-3.67835000	0.78108400	-0.21261600
C	-0.07372800	-1.33462000	-1.15802500	C	-0.08083400	-1.35142100	-1.14881800
Conformer 4 in DMSO				Conformer 4 in ethanol			
O	-2.87302100	2.94573800	0.79188100	O	-2.89208000	2.95749300	0.75298300
C	-3.10284700	-1.79206100	0.26336900	C	-3.10438600	-1.78667200	0.27883500
H	-3.91179000	-2.46218000	-0.03054300	H	-3.91117300	-2.46181800	-0.00963000
O	0.17022800	-1.54859600	-2.15911400	O	0.19728300	-1.62024100	-2.12485500
C	-1.82545700	-2.30234800	-0.43266300	C	-1.82385700	-2.30344800	-0.40618000
H	-1.67194400	-3.35203400	-0.15470500	H	-1.66968400	-3.34970000	-0.11520900
H	-1.91426800	-2.24781800	-1.51339500	H	-1.90950900	-2.26289200	-1.48779800
O	0.46786600	1.58067100	-0.27619700	O	0.46404100	1.56171100	-0.25866100
N	-0.65400900	-1.51854600	-0.04026900	N	-0.65550200	-1.51180200	-0.02033100
C	-0.47302900	-1.33599000	1.40167100	C	-0.48062400	-1.30782900	1.41958200
H	0.33448200	-0.63097000	1.57874000	H	0.32291400	-0.59675800	1.58969400
H	-0.18040600	-2.29251800	1.85144100	H	-0.18525400	-2.25676100	1.88387100
C	-1.76143800	-0.84845800	2.08267600	C	-1.77265200	-0.81676300	2.09093800
H	-1.55434400	-0.83190000	3.15504800	H	-1.56847600	-0.78723200	3.16365200
C	-2.16406100	0.57553200	1.69354200	C	-2.17960300	0.60108200	1.68484100
H	-2.74635500	1.02642300	2.49910200	H	-2.76847900	1.05752200	2.48258900
H	-1.29174700	1.20922300	1.54309900	H	-1.30977600	1.23757300	1.53237600
N	-2.98698600	0.68075000	0.46555200	N	-2.99607400	0.68881100	0.45155700
C	-3.29585600	2.01379100	0.09998800	C	-3.30693600	2.01687700	0.06877800
C	-4.10559800	2.16109600	-1.07524700	C	-4.10945900	2.14760600	-1.11356000
H	-4.33346400	3.17433500	-1.37743600	H	-4.33786500	3.15650900	-1.42948300
C	-4.57484500	1.07629500	-1.75269600	C	-4.57147600	1.05344400	-1.78059700
H	-5.19739400	1.21106200	-2.63007500	H	-5.18867400	1.17588100	-2.66357000
C	-3.47064800	-0.40508700	-0.21454400	C	-3.47286700	-0.40634500	-0.21801500

C	-4.26407100	-0.22726700	-1.31623200	C	-4.26022700	-0.24405300	-1.32634700
H	-4.64178100	-1.09537100	-1.83788700	H	-4.63235100	-1.11942800	-1.83984300
C	-2.90977200	-1.81068600	1.78055700	C	-2.91688700	-1.78632500	1.79677800
H	-2.67059000	-2.82195900	2.11764300	H	-2.67597600	-2.79277900	2.14702600
H	-3.82341200	-1.49991100	2.29273600	H	-3.83325400	-1.47211300	2.30201800
O	2.66247700	1.55246700	0.00435100	O	2.65999200	1.54603200	0.01238700
C	1.47485000	0.90962900	-0.27379900	C	1.47407500	0.89608700	-0.26030600
C	1.54495900	-0.51956900	-0.54437100	C	1.55211100	-0.53284100	-0.53193800
C	2.73689300	-1.15736700	-0.55125000	C	2.74912400	-1.16119600	-0.55054900
H	2.78637400	-2.21477900	-0.78712800	H	2.80373400	-2.21707500	-0.79190500
C	3.95400100	-0.46281400	-0.25406600	C	3.96324300	-0.45963700	-0.25882700
C	5.21859600	-1.07361200	-0.22777900	C	5.23200500	-1.06211400	-0.24151800
H	5.29396000	-2.13298600	-0.44168700	H	5.31323400	-2.12019800	-0.45970100
C	6.34634700	-0.33141000	0.06594500	C	6.35631600	-0.31355500	0.04894100
H	7.31887400	-0.80601000	0.08468700	H	7.33209400	-0.78166600	0.06085200
C	6.23195000	1.03643500	0.33925000	C	6.23417000	1.05252100	0.32780700
H	7.11744300	1.61581100	0.56894100	H	7.11700900	1.63692800	0.55502400
C	4.99593700	1.66365100	0.31920500	C	4.99403200	1.67165000	0.31633600
H	4.88922800	2.72031800	0.52654000	H	4.88137000	2.72687500	0.52779200
C	3.86896300	0.90772800	0.02250400	C	3.87038100	0.90943400	0.02276100
C	0.28574300	-1.23522200	-0.97763800	C	0.29806000	-1.26323700	-0.95461900

Table S2. Selected structural peculiarities of various conformers in DMSO and ethanol solutions.

Structural parameter	Conformer 1		Conformer 2		Conformer 3		Conformer 4	
**Bond, Å**	**DMSO**	**Ethanol**	**DMSO**	**Ethanol**	**DMSO**	**Ethanol**	**DMSO**	**Ethanol**
C2C27	1.530	1.530	1.530	1.530	1.530	1.530	1.530	1.529
C13C27	1.529	1.529	1.529	1.529	1.529	1.529	1.528	1.528
C2C5	1.547	1.547	1.543	1.543	1.546	1.546	1.542	1.541
C13C10	1.535	1.535	1.538	1.538	1.535	1.535	1.537	1.537
C5N9	1.458	1.458	1.462	1.462	1.461	1.461	1.463	1.463
C10N9	1.465	1.465	1.463	1.462	1.466	1.466	1.465	1.465
N9C45	1.357	1.357	1.357	1.357	1.357	1.358	1.357	1.358
C45O4	1.229	1.228	1.229	1.228	1.227	1.227	1.228	1.228
C45C32	1.510	1.510	1.510	1.510	1.509	1.509	1.512	1.512
**Angle,^{o}**								
C24C2C5	110.58	110.58	110.71	110.72	110.97	111.03	111.25	111.24
C15C13C10	113.30	113.30	113.23	113.23	113.29	113.3	113.78	113.82
C2C5N9	110.81	110.82	111.24	111.25	111.16	111.19	111.41	111.39
C13C10N9	111.83	111.83	111.50	111.51	111.83	111.82	111.85	111.94
C5N9C10	115.18	115.19	115.34	115.36	115.18	115.23	115.45	115.46
C5N9C45	125.14	125.16	119.43	119.40	125.41	125.39	118.75	118.58
C10N9C45	119.64	119.62	125.15	125.16	119.37	119.33	124.64	124.54
N9C45O4	123.13	123.11	123.18	123.16	123.36	123.36	123.11	123.02
N9C45C32	118.41	118.44	118.31	118.32	118.54	118.54	118.53	118.60
C32 C45O4	118.46	118.45	118.51	118.52	118.09	118.09	118.36	118.37
C45C32C31	118.46	118.56	118.27	118.33	116.26	116.24	118.54	118.80
**Dihedral Angle,^{o}**								

N18C15C13C27	-39.17	-39.14	-39.05	-39.04	-37.99	-37.78	-37.21	-37.40
N18C24C2C27	32.05	32.06	32.07	32.09	32.37	32.34	31.19	31.06
C15C13C10N9	-67.66	-67.64	-68.09	-68.10	-67.59	-67.59	-68.09	-68.25
C24C2C5N9	65.54	65.56	65.59	65.62	66.15	66.24	66.64	66.59
C2C5N9C10	51.56	51.54	51.39	51.36	50.87	50.76	50.01	49.93
C13C10N9C5	-50.96	-50.96	-50.29	-50.26	-50.52	-50.46	-49.66	-49.50
C13C10N9C45	131.14	131.09	132.93	133.12	131.59	132.02	142.87	144.33
C2C5N9C45	-130.68	-130.64	-131.63	-131.82	-131.39	-131.89	-141.74	-143.03
C10N9C45O4	-5.82	-5.96	-177.17	-177.21	-3.28	-3.14	170.81	169.18
C33C32C45N9	119.08	119.76	-118,64	-119.02	-78.50	-79.53	111.10	113.66
N9C45C32C31	-68.39	-67.76	68.69	68.33	108.71	107.71	-76.14	-73.62
O4C45C32C31	111.96	112.64	-111.58	-111.98	-72.44	-73.36	104.83	107.33
Hydrogen contacts					3.394 (O4H12)	3.387 (O4H12)	3.266 (O4H6)	3.243 (O4H6)
					2.275 (O4H11)	2.275 (O4H11)	2.292 (O4H7)	2.292 (O4H7)
							2.558 (O8H17)	2.541 (O8H17)
	2.277 (O4H11)	2.276 (O4H11)	2.275 (O4H7)	2.274 (O4H7)				
	2.996 (O8H6)	2.980 (O8H6)	2.948 (O8H12)	2.934 (O8H12)				
	2.902 (O8H7)	2.887 (O8H7)	2.931 (O8H11)	2.921 (O8H11)				

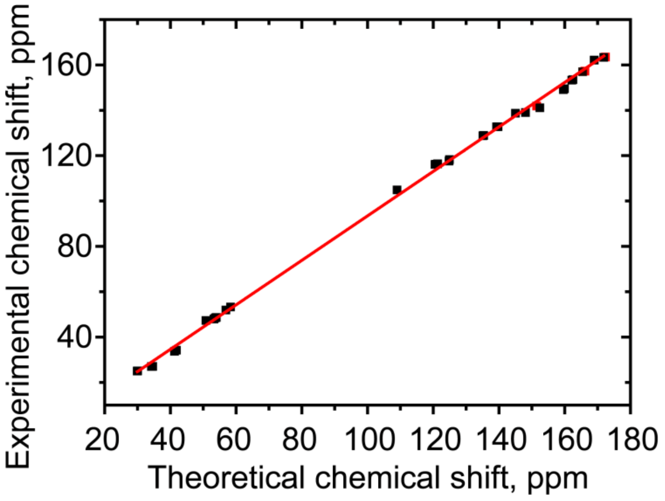

Figure S5. The linear regression between theoretical unscaled and experimental 13C NMR chemical shifts for the selected signals of the conformer 2 and non-overlapping signals of conformer 1. For linear regression the intercept value is -4.647±0.710, the slope is 0.981±0.006. The Pearson's r is 0.999. To

construct this dependence, individual values were selected that are interpreted unambiguously and correspond to well-resolved signals.

Table S3. Interpretation of the 13C NMR spectrum of complex (the chemical shifts are given unscaled as calculated with respect to the TMS 13C signal).

**Experimental chemical shifts for the complex, ppm**	**Experimental chemical shifts for the** *coumarin** **and cytisine ** molecules, ppm**	**Theoretical chemical shift, ppm**				**Assignment**
		**Conformer1**	**Conformer2**	**Conformer3**	**Conformer4**	
163.49	--	--	--	171.2555	172.4117	45-C
163.32	--	171.8866	171.8443	--	--	45-C
162.15	**163.4**	--	--	169.0174	169.0144	19-C
162.06	**163.4**	168.9653	168.982	--	--	19-C
157.27	*160.63*	--	--	--	166.1371	31-C
156.98	*160.63*	165.456	165.535	165.3719	--	31-C
153.49	*153.99*	162.5175	--	--	--	44-C
153.3	*153.99*	--	162.583	162.1754	162.4595	44-C
149.49	**150.9**	--	159.9177	--	160.1136	24-C
149.08	**150.9**	159.5802	--	159.5861	--	24-C
141.87	*143.48*	--	--	--	151.4823	33-C
141.08	*143.48*	152.4408	152.6098	--	--	33-C
139	**138.6**	--	--	148.066	--	33-C
138.68	**138.6**	144.9908	145.1984	145.008	144.8937	22-C
132.75	*131.79*	139.7217	139.744	--	139.6018	40-C
132.68	*131.79*	--	--	139.2715	--	40-C
128.91	*127.95*	--	135.4387	--	--	36-C
128.75	*127.95*	135.3449	--	135.1018	135.235	36-C
124.94	*116.56*	131.3091	131.6016	--	132.4418	32-C
124.89	*116.56*	--	--	--	130.4327	32-C
124.77	*124.43*	130.3224	130.3942	130.292	--	38-C
124.03	*124.43*	--	--	130.039	--	32-C
118.24	*118.81*	--	124.9409	--	124.9801	35-C
117.6	*118.81*	124.7923	--	124.4982	--	35-C
116.39	*116.70*	--	121.3753	--	121.434	42-C
116.29	*116.70*	121.3348	--	121.3614	--	42-C
116.22	**116.4**	120.5373	--	120.8463	120.5345	20-C
116.1	**116.4**	--	120.4147	--	--	20-C
104.87	**104.7**	109.0013	109.2664	108.9049	108.9065	25-C
53.25	**53.8**	58.3076	--	58.6411	--	5-C
51.89	**53.8**	--	56.929	--	56.7108	10-C
48.64	**52.8**	54.0081	--	53.9949	--	15-C
48.42	**52.8**	--	53.6788	--	53.2571	15-C
47.92	**49.5**	--	52.0665	--	52.4991	5-C
47.28	**49.5**	50.831	--	50.7754	--	10-C
39.5	--	--	--	--	--	13C in DMSO

						solvent
34.16	**35.4**	41.8485	--	41.7332	--	2-C
33.7	**35.4**	--	41.2832	--	40.9694	2-C
27.05	**27.5**	--	34.6485	--	--	13-C
26.96	**27.5**	34.2509	--	34.2468	34.2464	13-C
25.06	**26.1**	30.0774	--	30.027	--	27-C
24.99	**26.1**	--	29.9435	--	29.9145	27-C

*data taken from the AIST database (SDBS №802) [22] is highlighted in bold;

** data taken from [20] is highlighted in italics.

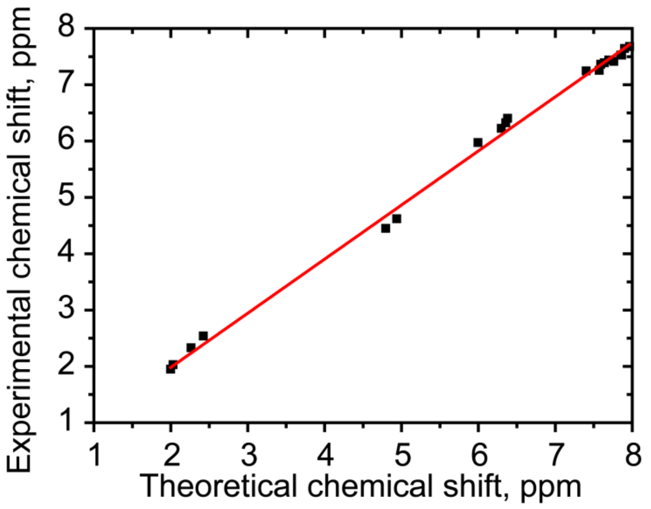

Figure S6. The linear regression between theoretical unscaled and experimental 1H NMR chemical shifts for the selected signals of the conformer 2 and non-overlapping signals of conformer 1. For linear regression the intercept value is 0.061±0.003, the slope is 0.961±0.004. The Pearson's r is 0.999. To construct this dependence, individual values were selected that are interpreted unambiguously and correspond to well-resolved signals.

Table S4. Interpretation of the 1H NMR spectrum of complex (the chemical shifts are given scaled with respect to the TMS 13C signal).

**Experimental chemical shifts for the complex, ppm**	**Experimental chemical shifts for the** *coumarin** **and (-)-cytisine** molecules, ppm**	**Theoretical chemical shift, ppm**				**Assignment**
		**Conformer1**	**Conformer2**	**Conformer3**	**Conformer4**	
7.67-7.69	*7.532*	7.9666	7.9886	7.9497	7.988	41-H
7.65-7.64	*7.727*	--	7.9011	--	7.9716	34-H
7.53-7.52	*7.498*	--	7.8613	--	7.893	37-H
7.53-7.52	*7.498*	7.7608	--	7.8513	--	37-H
7.45-7.43	*7.727*	7.6968	--	7.6681	--	34-H

7.42-7.38	*7.285*	7.6381	7.6743	7.6545	7.6813	39-H
7.37-7.36	*7.320*	7.5916	7.6193	7.5472	7.6241	43-H
7.25-7.26	**7.30**	--	7.5714	--	7.5446	23-H
7.25-7.24	**7.30**	7.4012	--	7.4753	--	23-H
6.41-6.4	**6.45**	--	6.379	6.3737	6.3627	21-H
6.32-6.3	**6.45**	6.3563	--	--	--	21-H
6.23-6.22	**6.00**	--	6.2992	--	6.2667	26-H
5.98-5.97	**6.00**	5.9949	--	6.0591	--	26-H
4.63-4.61	**3.02**	4.9386	--	4.9719	--	11-H
4.46-4.44	**3.08**	--	4.7954	--	4.6996	7-H
3.98-3.85	**4.13**	4.0737	3.8703	4.033	4.147	17-H
3.34-3.72	**3.13**	3.0273	3.5766	3.0186	3.3632	12-H
3.34-3.72	**3.89**	3.6244	3.5186	3.6546	3.6471	16-H
3.34-3.72	**3.02**	--	3.5593	--	3.5387	11-H
3.34-3.72	**3.08**	3.4661	--	3.7798	--	7-H
3.34-3.72	**3.02**	3.6047	--	3.5697	--	6-H
3.22-3.05	**2.91**	2.8979	3.0532	2.9038	3.0672	3-H
3.22-3.05	**3.02**	--	3.0659	--	3.0528	6-H
2.54	**2.35**	2.4231	--	2.4492	--	14-H
2.5	--	--	--	--	--	Residual 1H in DMSO solvent
2.33	**2.35**	--	2.2645	--	2.2961	14-H
2.03	**1.96**	2.0295	2.0249	2.0221	1.9729	28-H
1.95	**1.96**	1.9983	1.9967	1.9917	1.9783	29-H

*data taken from the AIST database (SDBS №802) [22] is highlighted in italics;

** data taken from [23] is highlighted in bold.

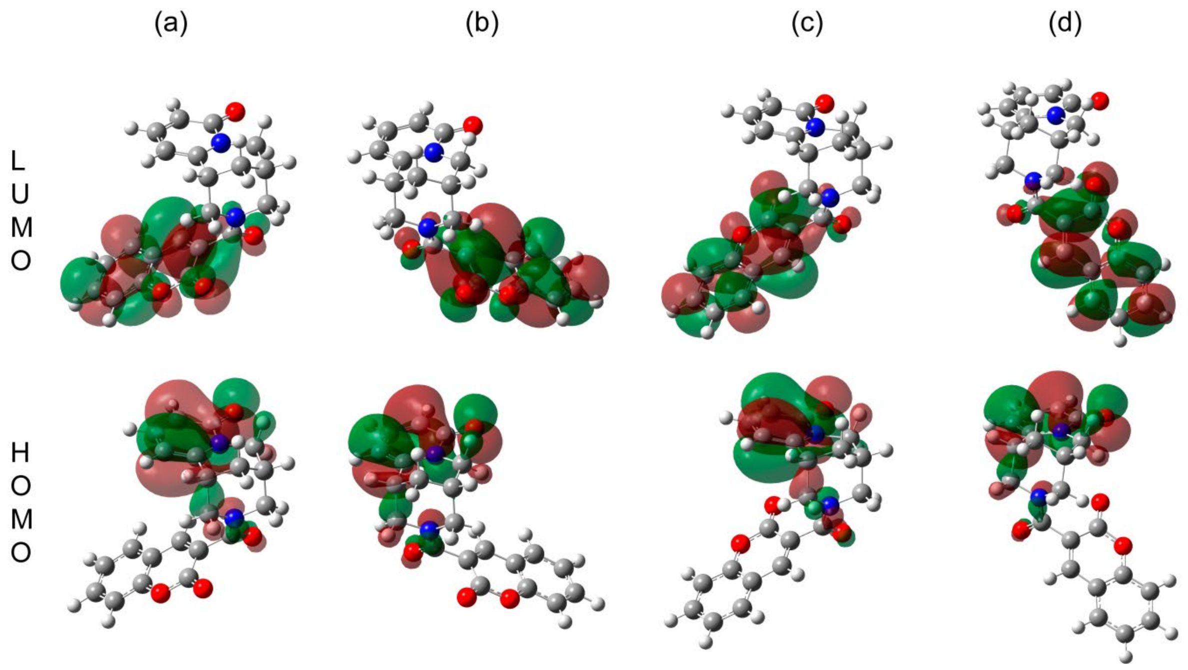

Figure S7. HOMO and LUMO orbitals for conformers 1 (a), 2 (b), 3 (c) and 4 (d).

References

20. Przybył, A.K.; Janczak, J.; Huczyński, A. Synthesis and Structural Analysis of New (−)-Cytisine Squaramides. *Molecules* **2025**, *30*, 1135. https://doi.org/10.3390/molecules30051135
22. AIST database SDBS #802 Available online: https://sdbs.db.aist.go.jp/ (accessed on 31 August 2025).
23. Berger S.; Sicker D. *Classics in spectroscopy: Isolation and Structure Elucidation*; John Wiley & Sons, Weinheim, Germany, 2009, pp. 659
